# Night‐time warming in the field reduces nocturnal stomatal conductance and grain yield but does not alter daytime physiological responses

**DOI:** 10.1111/nph.19075

**Published:** 2023-07-10

**Authors:** Lorna McAusland, Liana G. Acevedo‐Siaca, R. Suzuky Pinto, Francisco Pinto, Gemma Molero, Jaime Garatuza‐Payan, Matthew P. Reynolds, Erik H. Murchie, Enrico A. Yepez

**Affiliations:** ^1^ Division of Plant and Crop Sciences, School of Biosciences University of Nottingham Leicestershire LE12 5RD UK; ^2^ International Maize and Wheat Improvement Centre (CIMMYT) Carretera México‐Veracruz Km 45, El Batán, Texcoco México CP 56237 Mexico; ^3^ Instituto Tecnológico de Sonora (ITSON) 5 de Febrero 818 Sur, Col. Centro, Cd. Obregón, Sonora México CP 85000 Mexico

**Keywords:** night, nocturnal, respiration, stomatal conductance, temperature, T‐FACE, wheat, yield

## Abstract

- Global nocturnal temperatures are rising more rapidly than daytime temperatures and have a large effect on crop productivity. In particular, stomatal conductance at night (*g*_sn_) is surprisingly poorly understood and has not been investigated despite constituting a significant proportion of overall canopy water loss.
- Here, we present the results of 3 yr of field data using 12 spring *Triticum aestivum* genotypes which were grown in NW Mexico and subjected to an artificial increase in night‐time temperatures of 2°C.
- Under nocturnal heating, grain yields decreased (1.9% per 1°C) without significant changes in daytime leaf‐level physiological responses. Under warmer nights, there were significant differences in the magnitude and decrease in *g*_sn_, values of which were between 9 and 33% of daytime rates while respiration appeared to acclimate to higher temperatures. Decreases in grain yield were genotype‐specific; genotypes categorised as heat tolerant demonstrated some of the greatest declines in yield in response to warmer nights.
- We conclude the essential components of nocturnal heat tolerance in wheat are uncoupled from resilience to daytime temperatures, raising fundamental questions for physiological breeding. Furthermore, this study discusses key physiological traits such as pollen viability, root depth and irrigation type may also play a role in genotype‐specific nocturnal heat tolerance.

## Introduction

Climate change is driving rapid increases in global ambient temperature, particularly at night where minimum (*T*_min_) night‐time temperatures are rising at 1.4× the rate of daytime temperatures (Davy *et al*., 2017; Sadok & Schoppach, 2019). While episodic daytime heatwaves are either chronic (long‐term) or acute (short‐term), high nocturnal temperatures often extend over long periods of crop cycles and encompass large geographical areas leading to negative impacts on phenology and yield. These negative impacts have been reported for major crop species like rice (*Oryza* spp. – Peng *et al*., 2004; Welch *et al*., 2010; Coast *et al*., 2014; Shi *et al*., 2016; Bahuguna *et al*., 2017; Bheemanahalli *et al*., 2021), barley (*Hordeum vulgare*, García *et al*., 2015, 2016), sorghum (*Sorghum bicolor*, Prasad *et al*., 2011), soybean (*Glycine max*, Lin *et al*., 2021), quinoa (*Chenopodium quinoa* – Lesjak *et al*., 2017) and cotton (*Gossypium hirsutum*, Loka *et al*., 2010).

This penalty is particularly evident in wheat, with Spring wheat yields reported to decline 3.2–8.4% for every 1°C increase in *T*_min_ (Lobell *et al.*, 2005; Lobell & Ortiz‐Monasterio, 2007) or 4% decline for every 1°C increase over 14°C (Fischer, 1985). High nocturnal temperatures have been shown to decrease wheat seed set, grain number and grain yield between 13 and 43% (Narayanan *et al*., 2015). This fall in yields has been attributed to rapid, premature depletion of photoassimilates for growth due to increases in respiration (García *et al*., 2016), a shortening of the grain‐filling period and/or reductions in pollen fertility (Narayanan *et al*., 2018) leading to fewer grains setting.

While daytime heat tolerance mechanisms have been studied extensively (for a review; Moore *et al*., 2021), nocturnal processes have received less attention, especially under field conditions. One of the consequences of rising nocturnal temperatures is the significant alteration of whole‐plant water use, either through reduction in available water at the roots or through changes in stomatal behaviour via increased [CO_2_], changes in water potential or increased vapour pressure deficit (VPD). While daytime water loss via the stomata (*g*_s_ *–* please refer to Box 1 for all abbreviations used in this manuscript) can be correlated with the exchange of CO_2_ for photosynthetic carbon assimilation (*A*), recent work has shown that stomata open at night in wheat (McAusland *et al*., 2021). However, a definitive role for nocturnal conductance (*g*_sn_) has remained elusive, especially in the context of nocturnal warming. Nocturnal conductance typically accounts for 5–18% of all daytime water loss in wheat (Caird *et al*., 2007; McAusland *et al*., 2021) and is growth stage and genotypic‐specific. Nocturnal stomatal conductance has been correlated with the breakdown of starch (dos Anjos *et al*., 2018), maintenance of growth (Fricke, 2019), and as a potential mechanism for facilitating the uptake of O_2_ and, in some cases, the release of respiratory CO_2_ (Daley & Phillips, 2006; Resco de Dios *et al*., 2016; Even *et al*., 2018; Fricke, 2019). To date, no study has dissected the response of wheat *g*_sn_ in the context of nocturnal heating in the field.

Box 1 **List of commonly utilised abbreviations referred to in this article.**

**Table:** 

Abbreviations	Units	Description
T‐FACE	NA	Temperature Free‐Air Controlled Enhancement
*T*_min_	°C	Minimum ambient temperature
*T*_max_	°C	Maximum ambient temperature
*g*_s_	mol m^−2^ s^−1^	Daytime stomatal conductance
*g*_sn_	mol m^−2^ s^−1^	Nocturnal stomatal conductance
*A*	μmol m^−2^ s^−1^	Rate of photosynthetic CO_2_ assimilation
*R*_d_	μmol m^−2^ s^−1^	Rate of nocturnal respiration
WSC	g m^−2^	Water‐soluble carbohydrate content
Ψ	MPa	Leaf water potential
Ψ_PD_	MPa	Predawn leaf water potential
Ψ_M_	MPa	Midday leaf water potential
GY	g m^−2^	Grain yield
TGW	g	Thousand‐grain weight
BM	g m^−2^	Biomass
HI	%	Harvest index
GN	m^−2^	Grain number per m^−2^
SN	m^−2^	Spike number per m^−2^
GPS	#	Grains per spike
GWPS	g	Grain weight per spike
PH	m	Plant height
GFP	%	Grain‐filling period, the period between anthesis and maturity
GYPR	g m^−2^ d^−1^	Grain yield production rate
CGR	g m^−2^ d^−1^	Grain yield production rate

There is an urgency to study the responses of field‐grown wheat under elevated ambient temperatures at night to determine the ‘real‐world’ impact of nocturnal warming as climate change progresses (Kimball *et al*., 2008). Since the responses of photosynthesis and plant productivity have not widely been characterised under the context of elevated nocturnal temperatures, it is also imperative to characterise natural variation between genotypes to determine whether this response can be optimised in the future to cope with increased temperatures. First proposed by Harte & Shaw (1995), using an array of infrared heaters provides a means to artificially raise the surface temperature of canopy below without having to overcome changes in boundary layer, in effect a Temperature Free‐Air Controlled Enhancement (T‐FACE) set‐up. In addition, using canopy temperature sensors, the system can be closely modulated using feedback loops to maintain specific nocturnal temperature thresholds as the field environment fluctuates (Nijs *et al*., 1996; Kimball, 2005). T‐FACE experiments have previously been used to assess the effect of elevated temperature in C_3_ crops such as perennial ryegrass (*Lolium perenne*), white clover (*Trifolium repens* L.) and rice (*Oryza sativa*; Nijs *et al*., 1996; Zhang *et al*., 2019; Wang *et al*., 2022).

Here, 12 genotypes were selected based on previous evaluation of yield under heat tolerance and investigated for genotype‐specific variation in *g*_sn_ at two growth stages under control and a nocturnal heat treatment of +2°C above ambient. In this study, we aim to: characterise natural variation of photosynthetic traits, especially stomatal conductance both during the day and the night; assess the impact of nocturnal heat on stomatal conductance, respiration and leaf water potential; and evaluate the effect that elevated nocturnal temperatures have on overall yield and biomass production. We hypothesise that: *g*_sn_ will be genotype‐specific and decline with nocturnal warming; this will be accompanied by a rise in the rate of respiration in response to night‐time warming; daytime physiological measurements (e.g. leaf‐level CO_2_ assimilation) will demonstrate a decline under high night‐time temperatures; and, finally, we hypothesise that heat‐tolerant genotypes will also demonstrate greater yields under warmer nights when compared to genotypes with low heat tolerance.

## Materials and Methods

### Site, experimental treatments, irrigation regime and genotypes

#### Regular sowing

Three field experiments were carried out between 2020, 2021 and 2022, under fully irrigated yield potential conditions at the Norman E. Borlaug experimental station, Ciudad Obregon, Sonora, Mexico (27.33°N, 109.09°W). The soil composition is as described in Sayre *et al*. (1997). The experiment consisted of two biological replicates under both the control (yield potential) and heated set‐up. Within each replicate, the 12 *Triticum aestivum* genotypes were sown in 0.5 × 3.2 m plots (*n* = 2). Three to five technical replicates (individual plants) were sampled within these plots. Each bed had a 24 cm gap between rows and 56 cm between beds. Herbicides, fungicides and pesticides were applied to minimise the impact of biotic stresses. Nitrogen fertiliser (100–200 units) was applied at the beginning of February each year. Irrigation was supplied throughout the growing period every 10–14 d using a drip‐based system. The genotypes were selected on broad heat tolerance (Table 1). Phenology was scored using the Zadoks decimal scale (Zadoks *et al*., 1974), considering a phenophase when > 50% of the population demonstrated the related characteristics. This study focused on two key developmental phases: booting (Z4.5) and heading (Z5.5). For the measurements of water‐soluble carbohydrates, measurements were made at heading (Z5.5) and physiological maturity (Z8.7).

**Table 1:** List of the genotypes investigated and their broad tolerance to heat.

CIMMYT GID	Cross name	Type	Heat tolerance
5893282	WEEBILL1	Bread	Low
3823821	PASTOR//HXL7573/2*BAU	Bread	Low
5180627	CMSS96Y04084S‐0Y‐1B‐131TLA‐0B‐0Y‐125B‐0Y‐0B	Bread	Low
7171118	SAUL or SAUAL/WHEAR//SAUAL/3/PBW343*2/KUKUNA*2//FRTL/PIFED	Bread	Low
5077000	CIRNO C 2008 or CIRNO	Durum	Intermediate
7806808	BORLAUG100 F2014	Bread	Intermediate
2465	PAVON F 76 or Pavon76	Bread	High
3825355	SOKOLL	Bread	High
3855011	VOROBEY	Bread	High
7129721	SOKOLL//PUB94.15.1.12/WBLL1	Bread	High
5865670	CMSS96Y04084S‐0Y‐1B‐93TLA‐0B‐0Y‐106B‐0Y‐0Y‐0Y‐0Y	Bread	High
6692380	PUB94 or PUB94.15.1.12/FRTL/5/CROC_1/AE.SQUARROSA (205)//BORL95/3/PRL/SARA//TSI/VEE#5/4/FRET2	Bread	High

The genotypes selected for this study, including their genotype identification numbers (GIDs), cross names, type and their broad tolerance to heat based on previous evaluations utilising CIMMYT germplasm that has been sown over several field seasons under yield potential and heat stress conditions (M. P. Reynolds & G. Molero, unpublished).

#### Late sowing

In 2021, data were collected from a late‐sown trial at heading where all 12 genotypes were sown on 26 January 2021, *c*. 5 wk later than the regular sowing. Drip irrigation was applied every 10–15 d.

### Nocturnal temperature treatment

Twenty‐one heaters (FTE‐1000 model, Comstock Park, MI, USA) were placed along the perimeter of the heat treatment blocks (Kimball, 2005) on a metal structure of 7.1 × 7.1 m surrounding each of the two heated blocks (Supporting Information Fig. S1) at 1.2 m above the canopy to maintain +2°C higher than the canopy temperature of the control blocks. Four infrared temperature sensors were placed in the corners of each of the four blocks (IRTS Apogee Instruments Inc., Logan, UT, USA) at an inclination of 45°. These sensors relayed temperature on the proportional integral derivative (PID) algorithm formulated in Kimball (2015), Kimball *et al*. (2015). Data were logged every 15 min (CR1000; Campbell Sci Inc., Logan, UT, USA).

### Crop measurements

#### Water potential

Leaf water potential (Ψ) was measured using a pressure chamber (Model 610; PMS instruments, Corvallis, OR, USA) following the method of Argentel‐Martínez *et al.* (2019). Two or three flag leaves per plot were harvested and measured at predawn (Ψ_PD_) and midday (Ψ_M_).

#### Water‐soluble carbohydrates

To determine the concentration of water‐soluble carbohydrates (WSCs), 12 fertile stalks (including leaves, leaf sheaths and heads, but not senesced plant material) were randomly sampled from each plot at heading and physiological maturity. The spikes were removed, and culms dried at 60–75°C until constant weight. Once dry, the leaf lamina and sheath were discarded. After measuring stem dry weight, samples were ground in a mill through a 0.5 mm screen and analysed using the Anthrone method (Yemm & Willis, 1954).

#### Flag leaf nitrogen and carbon content

Three flag leaves were collected at heading in the 3 yr, oven‐dried at 65°C for 48 h and ground to fine powder. Total nitrogen and carbon was estimated by flash combustion using an elemental analyser (Flash 2000; Thermo Scientific, Waltham, MA, USA).

#### Porometry

Porometers (Table S1, Li‐600; Li‐Cor, Lincoln, NE, USA) were used to measure the response of *g*_s_ and *g*_sn_ on the adaxial (top) and abaxial (bottom) flag leaf surfaces. Measurements were taken in 2021 and 2022 only. For 2020, this equipment was not available. For *g*_s_, measurements were taken across 3–4 clear, sunny days between 9:30 h−24:00 h and < 7 d after irrigation. For *g*_sn_, measurements occurred 30 min after sunset (*c*. 19:00 h) for a period of 1–2 h before dewfall. Nocturnal measurements were made on the same days that diurnal measurements were conducted. Two to three measurements were taken for the adaxial and abaxial leaf surfaces of the same leaves. Four to six measurements were made per genotype per treatment (*n =* 2 biological replicates).

#### Gas exchange

Measurements of *A*, *g*_s_, *R*_d_ and *g*_sn_ were taken using infrared gas analysers (LI‐6400‐XT; Li‐Cor). For daytime measurements, survey‐style measurements were conducted on flag leaves under 1800 μmol m^−2^ s^−1^ photosynthetic photon flux density (PPFD), using a 2 cm^2^ leaf chamber with a blue–red LED light source. Leaf temperature and VPD were maintained at 26°C (±1°C) and 1.2 kPa, respectively, while extracellular CO_2_ concentration (*C*_a_) was maintained at 400 μmol mol^−1^. At night, leaf temperature was 16°C (±1°C), while leaf VPD and *C*_a_ were kept the same as during the day. Measurements were taken as soon as values stabilised (1–2 min). Two to three measurements were made per genotype per treatment (*n =* 2 biological replicates), at booting and heading across all 3 yr of measurement (2020–2022).

#### Yield components and final yield

Grain yield (GY) was evaluated after genotypes had reached harvest maturity. Yield components were also measured: 1000‐grain weight (TGW), harvest index (HI), final aboveground biomass (BM), grain number (GN), spike number (SN), grain per spike (GPS), grain weight per spike (GWPS), grain‐filling period (GFP), grain‐filling rate (GFR), plant height (PH), grain yield production rate (GYPR = GY d^−1^) and crop growth rate (CGR = BM d^−1^). These evaluations follow the methodology of Pietragalla *et al*. (2012) and Pask *et al*. (2012). All traits related to yield and yield components were scaled on a per area basis for consistency.

### Statistical analyses

Data were analysed utilising a mixed linear model, accounting for year (2020–2022; fixed), treatment (heat, control; fixed), genotype (fixed), replicate/block (random), stage (booting, heading; fixed) and time (only for WP: predawn, midday; fixed) as factors. When applicable, individual plot, row and/or column were included as random factors. For the porometer data, leaf surface was also included as fixed factor. For the late‐sown material, overall parameter means were compared between the late‐sown data and regular‐sown heat and control plots. Mean discrimination between factors was determined utilising Tukey's honest significant difference (HSD). Means were compared through the Tukey's test at 95% of confidence. Visualisation and analysis of the data were performed in R (R Core Team, 2022).

## Results

### Field conditions

**Fig. 1:**
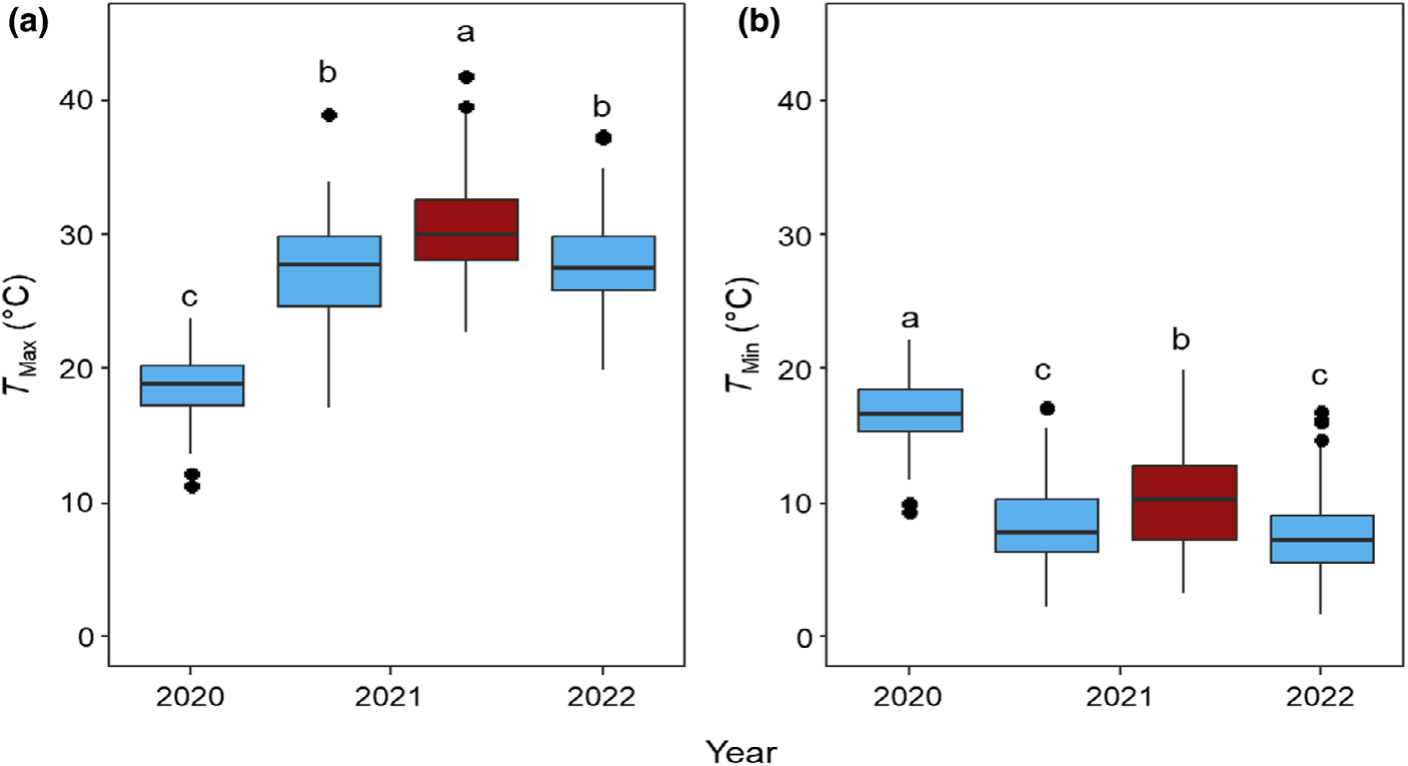
Average of the daily maximum (a – *T*_max_) and minimum air temperature (b – *T*_min_) during the growing season during all three field seasons (2019–2020, 2020–2021 and 2021–2022). All regular sowing experiments (blue) were sown on 14 or 13 December in 2019, 2020 and 2021. The late sowing experiment (dark red) during the 2021 field season was sown on 26 January 2021. Statistical differences are shown between boxplots as letters where the threshold for a significant difference is *P* < 0.05. The lower and upper borders of the boxplots correspond to the first and third quartiles of the data, the black lines within the boxes indicate the median. Outliers which fall outside the whiskers are shown as black dots (•).

Maximum (*T*_max_) and minimum air temperature (*T*_min_) averaged across the control and night heated treatments varied significantly across years. The regular sowing of 2020 experienced a significantly lower *T*_max_ while having a significantly higher *T*_min_ compared with 2021 and 2022 and the late sowing treatment in 2021 (Fig. 1). The two subsequent field seasons did not vary significantly for *T*_max_ or *T*_min_. The late sowing treatment during the 2021 field cycle was significantly (*P* < 0.0001) warmer for *T*_max_ across all years (Fig. 1).

### Conditions within T‐FACE set‐up and canopy temperature

The T‐FACE system maintained the canopies of the heated blocks at 2°C above the temperature of the control blocks. An average of 87% of nights of the season across the 3 yr of experiments recorded temperatures of > 1.8°C (±10%) above the temperature of canopies in the control blocks, with the remaining 13% reporting < 2°C increases probably due to high wind (Tables S2, S3). VPD increased minimally in the heated plots (Fig. S2), an increase of 0.07 kPa between the heat and controls across the 3 yr (Table S3).

While the average *T*_min_ of the nocturnally heated plots achieved 8.3°C for the measurement period in 2021, the late sowing plots achieved an average *T*_min_ of 10.3°C during the nights between booting and heading (Fig. 1).

### Crop measurements

#### Phenology

Phenology did not differ significantly (*P* = 0.12) between the control and nocturnally heated treatments across years. Heated plots were *c*. 2 d faster at reaching maturity (Table S4). Late sowing plots reached key phenological stages faster relative to both the control and nocturnally heated plots (Table S2).

#### Water potential

**Fig. 2:**
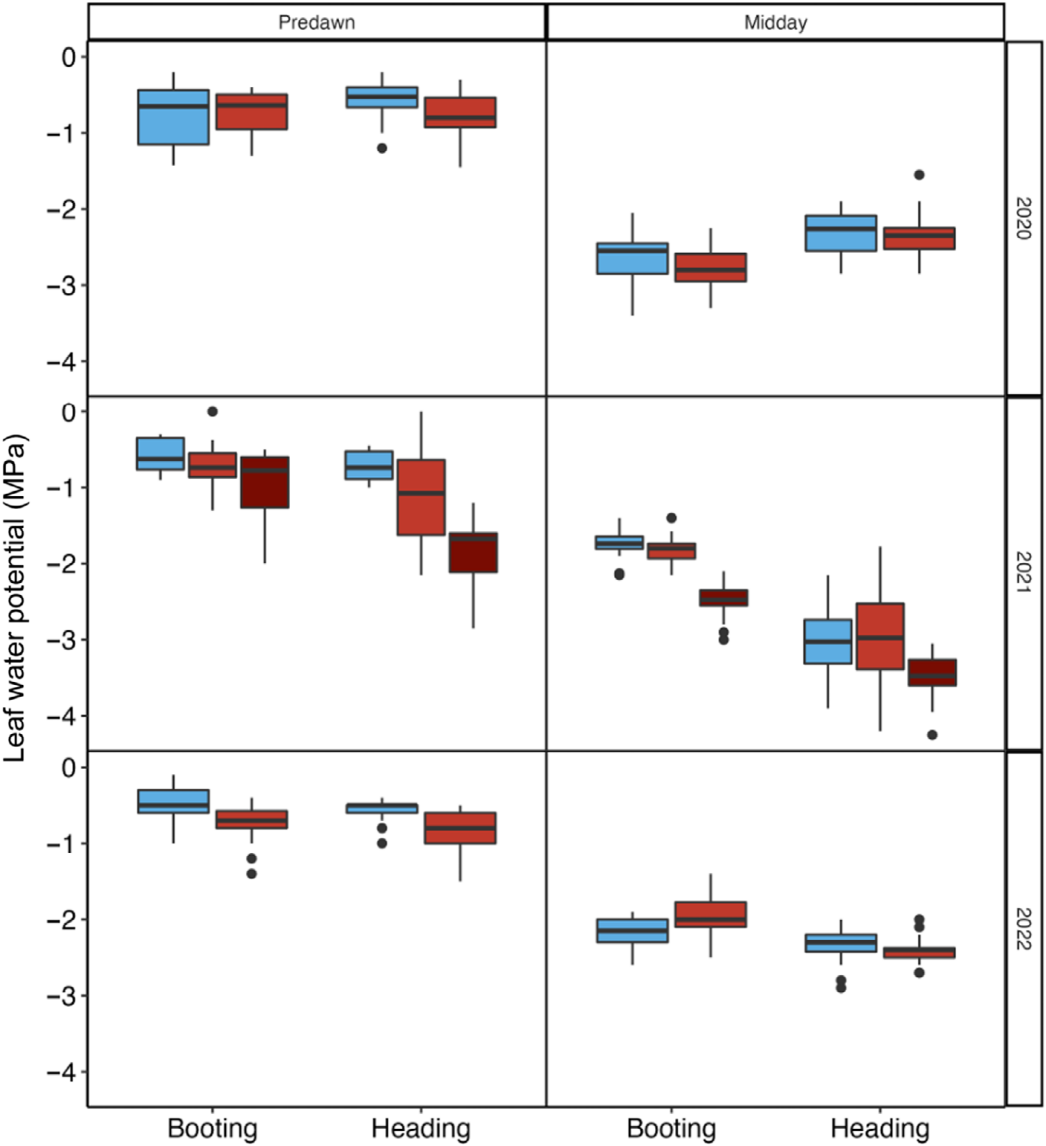
Response of leaf water potential measured predawn and at midday for leaves in booting or heading plants in 2020, 2021 and 2022. Regular‐sown plants were subject to control (blue) or nocturnal heating (light red). Measurements were also made on plants, which were late sown (dark red) in 2021. The data shown are the cumulative responses of 12 genotypes with two biological replicates grown in each treatment per year. The lower and upper borders of the boxplots correspond to the first and third quartiles of the data, and the black lines within the boxes indicate the median. Outliers which fall outside the whiskers are shown as black dots (•). Significant differences were determined using a mixed linear model (see ‘Statistical Analyses’ for further details).

Overall, water potential increased by up to 60% in response to nocturnal heating (Figs 2, S3); with the greatest increases observed in the predawn measurements at heading (−0.6 ± 0.2 MPa vs −0.9 ± 0.4 MPa, *P* < 0.0001). Significant differences between years were observed (*P* < 0.0001, Fig. S3). At heading, plants grown during 2021 (−0.9 ± 0.5 MPa) reported significantly higher Ψ_PD_ (*P* = 0.00070) than those grown in 2020 (−0.7 ± 0.3 MPa) and 2022 (−0.7 ± 0.2 MPa). Ψ_M_ was also significantly higher (28%, *P* < 0.0001) in the 2021 heading plants (−3.0 ± 0.5 MPa), compared with 2020 (−2.3 ± 0.3 MPa) and 2022 (−2.4 ± 0.2 MPa). Water potential became significantly more negative with age in both the control and nocturnally heated plots (*P* = 0.0014).

There were no genotype‐specific significant differences either predawn (*P* = 0.988; 2.5 ± 0.3 MPa) or at midday (*P* = 0.978; 0.7 ± 0.5 MPa) for the 3 yr of experiments under control or heated conditions at either growth stage. Ψ_PD_ was 2.9‐fold higher in the late sowing (1.8 ± 0.35 MPa) compared with the control plots from the regular sowing (−0.6 ± 0.19 MPa).

#### Porometry – *g*_sn_ and *g*_s_ variability by leaf surface

The increase of *g*_s_ and *g*_sn_ on the adaxial leaf surface was genotype‐specific but independent of year or treatment (*P* < 0.0001 for *g*_s_; *P* = 0.0199 for *g*_sn,_ Fig. S4). During the daytime, *g*_s_ was 95% higher on the adaxial surface (0.143 ± 0.12) compared with the abaxial (0.073 ± 0.06 mol m^−2^ s^−1^). Similarly, *g*_sn_ was 55% higher on the adaxial surface (0.013 ± 0.01) compared with the abaxial.

Mean *g*_sn_ for the adaxial leaf surface was only 11 and 13% higher than abaxial values at booting and heading, respectively (*P* > 0.05). Mean *g*_s_ and *g*_sn_ for the adaxial leaf surface were also significantly higher in the heated and in the control blocks when compared to the abaxial measurements (*P* < 0.0001, Table 2). The extent of the differences between leaf surfaces was highly dependent on treatment, showing that mean *g*_s_ and *g*_sn_ on the control blocks were 100% and 8% higher on the adaxial leaf surface compared with the abaxial measurements (*P* < 0.0001 and *P* = 0.355, respectively) and 85 and 26% higher on the heated blocks (*P* < 0.0001 and *P* = 0.012, respectively).

**Table 2:** Nocturnal stomatal conductance (*g*_sn_) as percentage of diurnal stomatal conductance (*g*_s_) as a function of treatment or leaf surface.

(a)					
Growth stage	Treatment	Mean *g*_sn_ (mol m^−2^ s^−1^)	Mean *g*_s_ (mol m^−2^ s^−1^)	*g*_sn_ as a % of g_s_	*P* value
Booting	Control	0.048	0.291	16.8 ± 1.07	< 0.0001
	Heat	0.026	0.287	8.8 ± 1.07	
Heading	Control	0.063	0.231	32.7 ± 1.35	0.007
	Heat	0.037	0.261	15.8 ± 1.32	

(b)					
Growth stage	Leaf surface	Mean *g*_sn_ (mol m^−2^ s^−1^)	Mean *g*_s_ (mol m^−2^ s^−1^)	*g*_sn_ as a % of g_s_	*P* value
Booting	Adaxial	0.036	0.363	10.4 ± 1.07	0.002
	Abaxial	0.032	0.214	15.1 ± 1.07	
Heading	Adaxial	0.052	0.341	16.1 ± 1.21	< 0.0001
	Abaxial	0.046	0.151	32.3 ± 1.41	

The increase of *g*_s_ and *g*_sn_ on the adaxial leaf surface was genotype‐specific but independent of year or treatment (*P* < 0.0001 for *g*_s_; *P* = 0.0199 for *g*_sn_). During the daytime, *g*_s_ was 95% higher on the adaxial surface (0.143 ± 0.12) compared with the abaxial (0.073 ± 0.06 mol m^−2^ s^−1^). Similarly, *g*_sn_ was 55% higher on the adaxial surface (0.013 ± 0.01) compared with the abaxial. For the late sowing, the magnitude of *g*_s_ and *g*_sn_ was also surface‐specific (*P* < 0.0001, *P* = 0.022, respectively).

Nocturnal stomatal conductance (*g*_sn_) as percentage of diurnal stomatal conductance (*g*_s_) averaged across 2 yr of experiments (2021 and 2022) under field conditions. Means and standard deviations for *g*_sn_ and *g*_s_ under treatments, stages and leaf surfaces are shown. The *P* value is indicated for the effect of (a) treatment or (b) leaf surface for each growth stage.

#### Gas exchange data

##### CO_2_ assimilation – *A*

Photosynthetic CO_2_ assimilation was significantly (*P* < 0.0001) higher in 2022 than in 2020 (+26.39%) and 2021 (+28.91%, Fig. S5). Nocturnal heating had a significant positive effect at booting in 2020 (+9.57%, *P* = 0.0062), but not at heading or at any growth stage in 2021 or 2022. *A* declined with age, falling between 19.93% (2022 – heated plots) and −43.47% (2020 – heated plots) between booting and heading. No significant interaction was determined between growth stage and heat treatment for 2021 or 2022. No significant differences were determined between genotype and treatment at booting (*P* > 0.08) or heading (*P* > 0.42) for any year.

##### Daytime stomatal conductance – *g*_s_

*g*_*s*_ was significantly (*P* < 0.0001) higher in 2022 than in 2020 (+79.25%) and 2021 (+55.00%, Fig. S6). The magnitude of *g*_s_ declining with age for all measurement years (*P* < 0.00127). Similar to *A*, there was only a significant interaction between treatment and growth stage in 2020 (*P* = 0.014). For all other measurement periods, *g*_s_ decreased −2.21% (2022 – heading) and −19.31% (2020 – heading) with nocturnal heating. Phenology had a greater, more consistent part to play in the decline of *g*_s_ (*P* < 0.007); up to 55.11% between booting and heading (2020 – heated). No significant differences were determined between genotype and treatment at booting (*P* > 0.668) or heading (*P* > 0.404) for any year.

##### Nocturnal respiration – *R*_d_

Respiration was significantly (*P* < 0.0001) higher in 2021 than in 2020 (−10.01%) and 2022 (−13.68%, Fig. S7). In general, *R*_d_ was expected to increase in response to heating and with age. However, these data suggest that respiration either remained unchanged or declined with heating – between 0.39 and 39.85% – and in response to age; 4.73–21.4%. 2021 was the only year where there was a significant, negative impact of heating at booting and heading; with *R*_d_ declining 39.85 and 34.99% when comparing the control and heated values under either booting or heading, respectively. There was no significant interaction between treatment and age (*P* < 0.123), but *R*_d_ significantly, consistently declined between both booting and heading in the control (*P* < 0.0001) and heated (*P* < 0.0001) plots.

##### Nocturnal stomatal conductance – *g*_sn_

**Fig. 3:**
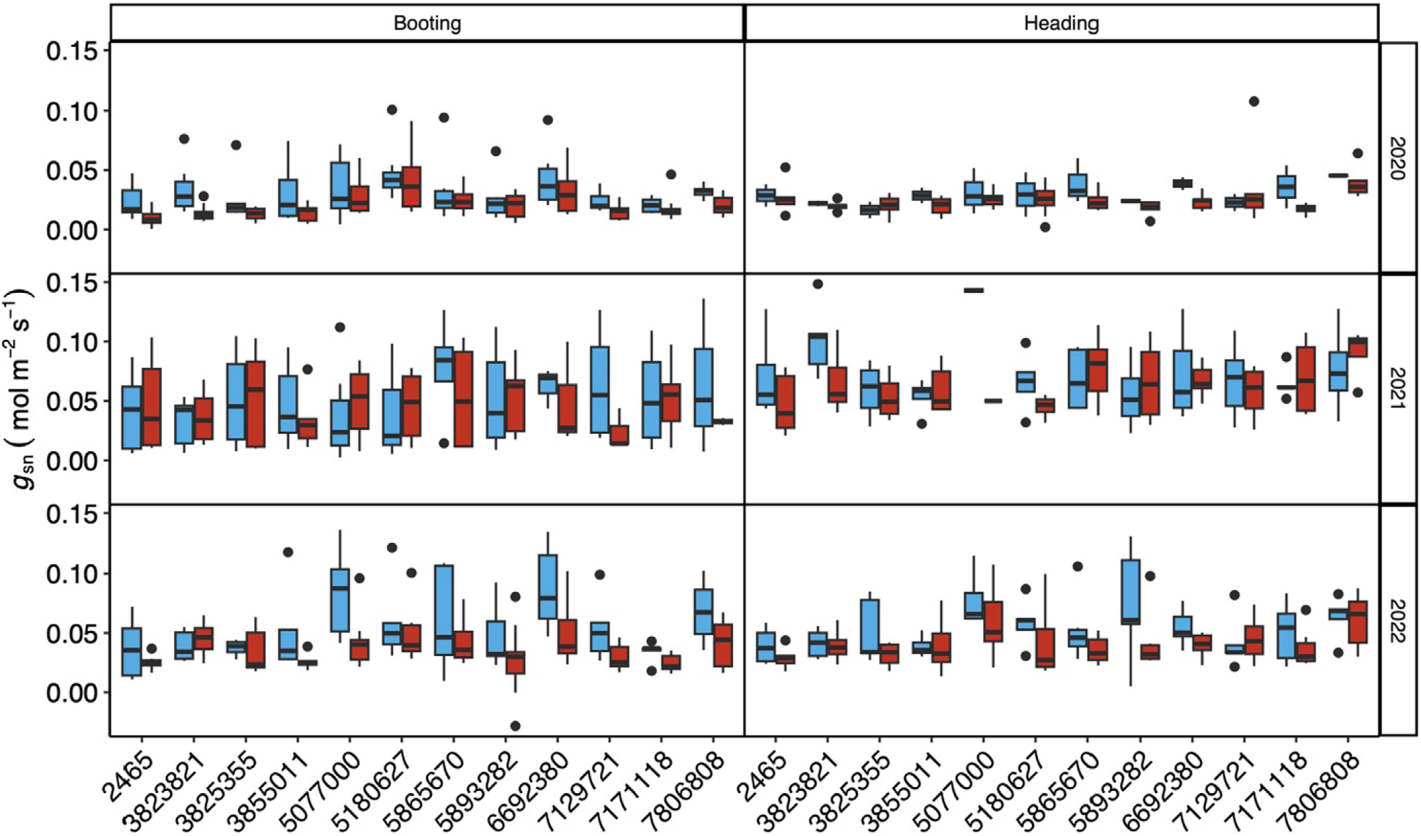
Response of nocturnal stomatal conductance (*g*_sn_) to control (blue) and nocturnal heating (red) of 12 genotypes (Table 1) measured utilising an infrared gas analyser. Measurements were taken at two growth stages over 3 yr of measurement (2020–2022) under regular sowing conditions. The lower and upper borders of the boxplots correspond to the first and third quartiles of the data, and the black lines within the boxes indicate the median. Outliers which fall outside the whiskers are shown as black dots (•). Significant differences were determined using a mixed linear model (see ‘Statistical Analyses’ for further details).

*g*_sn_ was significantly different between all years (*P* < 0.0001, Fig. S8). The lowest values were observed in 2020 (0.025), while the highest values were measured in 2021 (0.057). Measurements in 2022 were between these 2 yr at 0.046. There was no significant effect of heating in 2021 (*P* = 0.127); however, *g*_sn_ significantly declined between booting and heading (*P* < 0.0001). In 2020 and 2022, *g*_sn_ significantly declined in response to heat (*P* < 0.0201). There was no effect of growth stage in 2020 (*P* = 0.516) or 2022 (*P* = 0.225) and no interactions between growth stage and treatment for any of the measurement years (*P* > 0.216). Although there were significant genotype‐specific differences in 2020 and 2022 (Fig. S8, *P* < 0.003, booting), there was no interaction between the individual genotypes and nocturnal heat. (Fig. 3, *P* > 0.163).

##### Nocturnal observations

No one genotype consistently achieved the highest *R*_d_ or *g*_sn_ across growth stages and under control or heated conditions over all 3 yr of measurement. There was no trend between *R*_d_ and magnitude of *g*_sn_ under control (*P* = 0.306) or heated (*P* = 0.108) conditions.

##### The relationship between *g*_s_ and *g*_sn_

Irrespective of treatment or growth stage, there was a significant, weak positive correlation between *g*_s_ and *g*_sn_ – *P* = 0.0139 (*R*_adj_ = 0.035 – Fig. S9). When the data are separated by year, there is no trend between the magnitude of *g*_s_ and *g*_sn_, irrespective of treatment (Fig. S10).

Finally, *g*_sn_ as a proportion of *g*_s_ was calculated (Table 3). In general, *g*_sn_ made up a greater proportion of daytime *g*_s_ in the control plots and at heading. Heating led to a decrease in this proportion for both growth stages measured; however, this decrease was much greater at booting. There was a significant effect of heating (*P* = 0.0239) and growth stage (*P* < 0.0001) but no interaction between them (*P* = 0.7536).

**Table 3:** Mean nocturnal conductance (*g*_sn_) as a percentage of *g*_s_ at booting or heating under control or nocturnally heated plots.

Growth Stage	Treatment	*g*_sn_ as a % of *g*_s_
Booting	Control	12.30 ± 4.54
	Heat	9.38 ± 4.62
Heading	Control	19.40 ± 9.01
	Heat	17.2 ± 7.65

Mean nocturnal conductance (*g*_sn_) as a percentage of daytime stomatal conductance (*g*_s_) at booting or heating under control or nocturnally heated plots over the 3 yr. Data are the means (*n* = 6, biological replicates, with standard deviation).

##### Comparing the response of regular and late‐sown material

While no significant difference (*P* = 0.77) was determined between the regular‐sown plants, the late‐sown plants demonstrated significantly lower *A* (Fig. S11a) compared with the control (−59.0%) and heated plots (−60.4%). Similarly, *g*_s_ in the late‐sown plants exhibiting 76.6 and 75.2% declines compared with regular‐sown control (Fig. S11b, *P* < 0.0001) and nocturnally heated (*P* < 0.0001) plots.

There was no significant difference between the regular sowing treatments for *g*_s_ (*P* = 0.76). *R*_d_ was highest in the late‐sown plants (Fig. S11c), significantly higher than the plants under nocturnal heating (*P* < 0.0001) but not significantly different to the regular‐sown plants under control conditions. Finally, late sowing had a significant (*P* < 0.0001, Fig. S11d), negative impact on rates of *g*_sn_. Values were 71.4 and 68.0% lower than those observed in the regular‐sown control or nocturnally heated plots, respectively.

#### Water‐soluble carbohydrates, carbon and nitrogen content

**Fig. 4:**
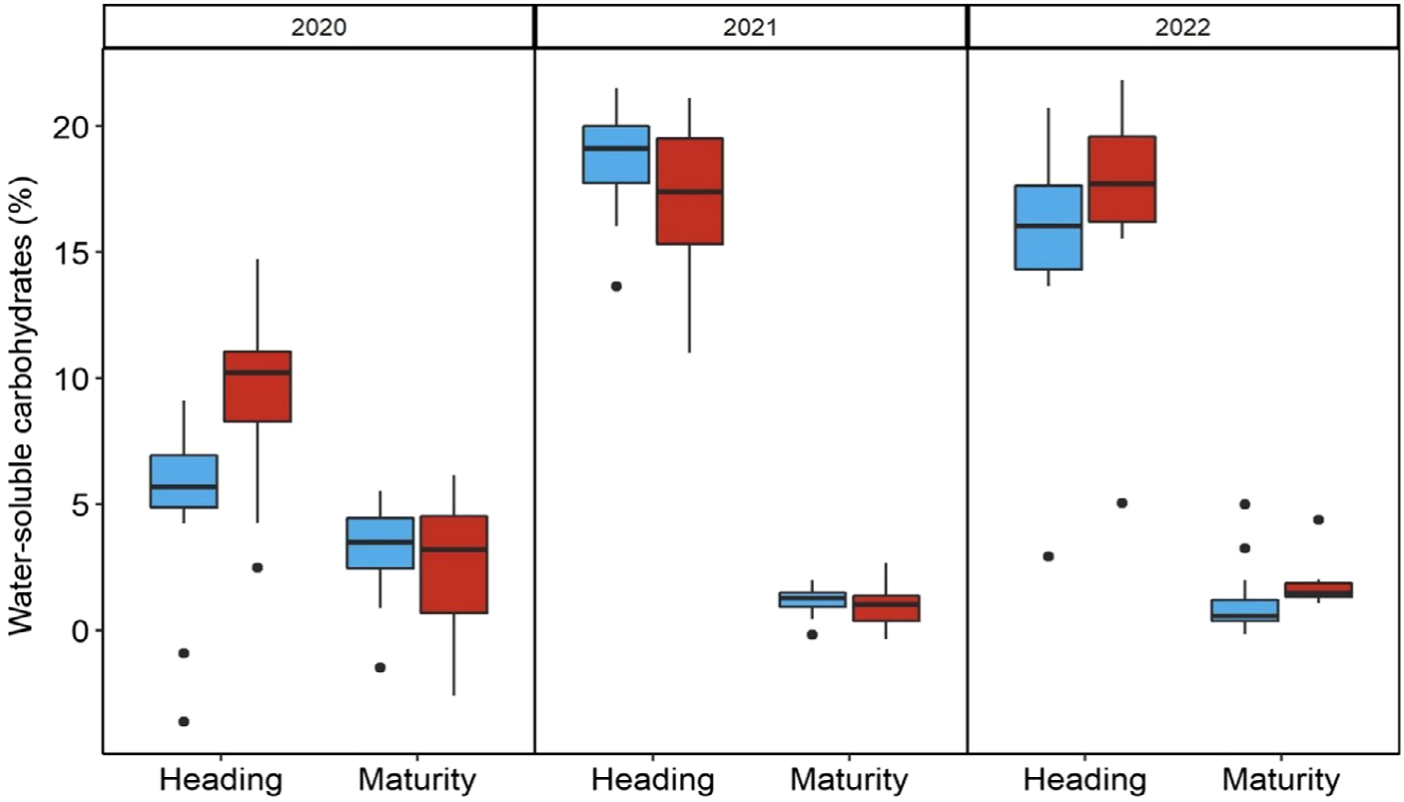
Average content of water‐soluble carbohydrates (WSCs) in control (blue) and nocturnally heated (red) plots. Wheat stems were sampled for the estimation of the relative content of WSC at heading and maturity over 3 yr of measurement (2020–2022) under regular sowing conditions. WSCs are expressed in percentage related to the dry matter of stems. The lower and upper borders of the boxplots correspond to the first and third quartiles of the data, and the black lines within the boxes indicate the median. Outliers which fall outside the whiskers are shown as black dots (•). Significant differences were determined using a mixed linear model (see ‘Statistical Analyses’ for further details).

Water‐soluble carbohydrates (WSC – Fig. 4) consistently decreased in all plots from heading to maturity. In 2020, the average WSC at heading was significantly lower (*c*. 7% to 14%, *P* < 0.05) than in 2021 and 2022. Conversely, at maturity, the average WSC in 2020 was highest (although not significantly for treated plots; *P* > 0.05). The nocturnal heat treatment resulted in higher concentration of WSC compared with control plants at heading in 2020 (4.4% higher, *P* < 0.05). A similar trend (*P* > 0.05) was observed during heading in 2022. WSC between treatments were not significant at maturity in any of the years.

**Fig. 5:**
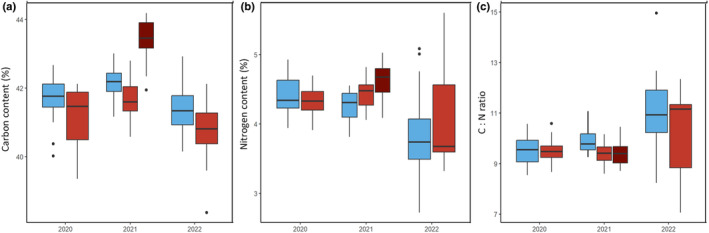
Average content of total flag leaf (a) carbon (%), (b) nitrogen (%) and (c) C : N ratio in the regular‐sown control (blue) and nocturnally heated (light red) plots across 12 heading genotypes over 3 yr (2020–2022). Content was also measured from leaves sampled from the 2021 late‐sown plots (dark red). The lower and upper borders of the boxplots correspond to the first and third quartiles of the data, and the black lines within the boxes indicate the median. Outliers which fall outside the whiskers are shown as black dots (•). Significant differences were determined using a mixed linear model (see ‘Statistical Analyses’ for further details).

Nocturnal heating consistently decreased the carbon content in the 3 yr (C – *P* < 0.001 – Fig. 5a); in 2021, the carbon content (41.9%) was significantly higher (*P* < 0.001) than in 2020 (41.4%) and 2022 (41.2%). The average carbon content in the heated and control plots was 41.2 and 41.8%, respectively. There were no genotypic differences (*P* > 0.05).

Significant differences were determined for flag leaf nitrogen content (N – Fig. 5b, *P* = 0.049) between the control (4.17 ± 0.44%) and the nocturnally heated plots (4.3 ± 0.50%). There was no significant interaction between measurement year and treatment and no significant effect of genotype (*P* > 0.05).

The C : N ratio was significantly (*P* = 0.00549 – Fig. 5c) higher in the control plots (10.1) compared with the nocturnally heated plants (9.7). Irrespective of treatment, plants measured in 2022 (10.6) had significantly higher ratios than those observed in 2020 (9.7) and 2021 (9.5, *P* < 0.0001). No significant interactions were determined between measurement year and treatment (*P* > 0.05). No significant genotypic effects were found (*P* > 0.05).

### Yield and yield component trends

**Fig. 6:**
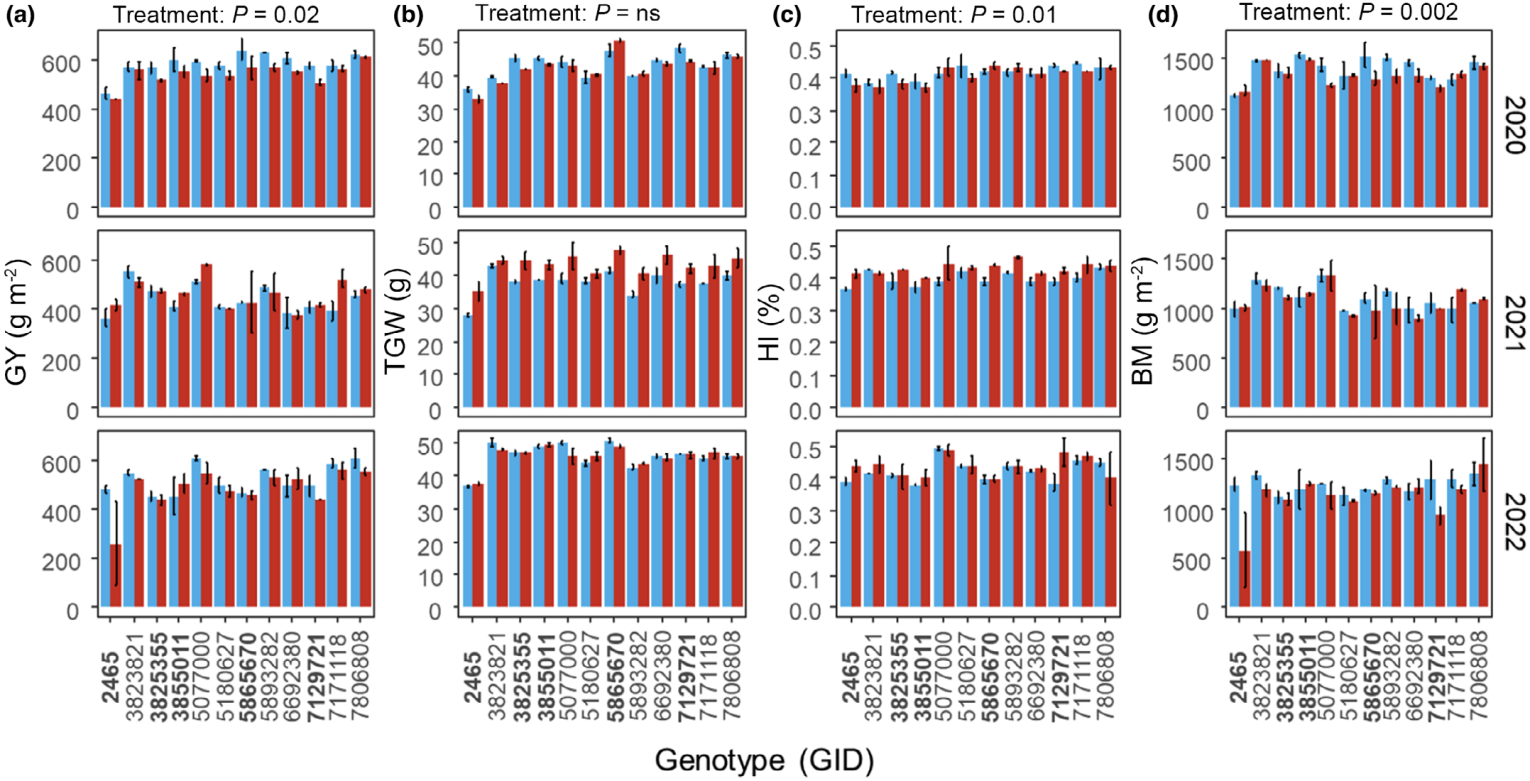
Variation in mean grain yield (a – GY), 1000‐grain weight (b – TGW), harvest index (c – HI) and final aboveground biomass (d – BM) across the three measured field seasons (2020, 2021 and 2022) in response to control conditions (blue) and nocturnal warming (red), between 12 genotypes. Genotype IDs in bold were predetermined as ‘heat tolerant’ (Table 1). Data are the means, while the error bars represent SE. Significant (*P* < 0.05) or nonsignificant (‘ns’) effect of treatment is indicated above each column. Significant differences were determined using a mixed linear model (see ‘Statistical Analyses’ for further details).

Combining the data across years, significant differences were found among genotypes for yield and yield component traits (Figs 6, S12, S13). The genotypes with the highest average yields across years in control conditions (Fig. 6a) were GID‐5077000 (576 g m^−2^) and GID‐7806808 (566.3 g m^−2^), while the highest under nocturnal heat were GID‐5077000 (557.8 g m^−2^) and GID‐7171118 (550.16 g m^−2^). The lowest yielding genotypes under control conditions were GID‐2465 (438.6 g m^−2^) and GID‐3855011 (488.6 g m^−2^), while under heat treatment it was GID‐2465 (373 g m^−2^) and GID‐7129721 (457.3 g m^−2^). Interestingly, GID‐2465, GID‐3855011 and GID‐7129721 are all considered high heat tolerance genotypes, while GID‐5077000 and GID‐7806808 are intermediate and GID‐7171118 is considered a low heat tolerance genotype (Table 1).

**Fig. 7:**
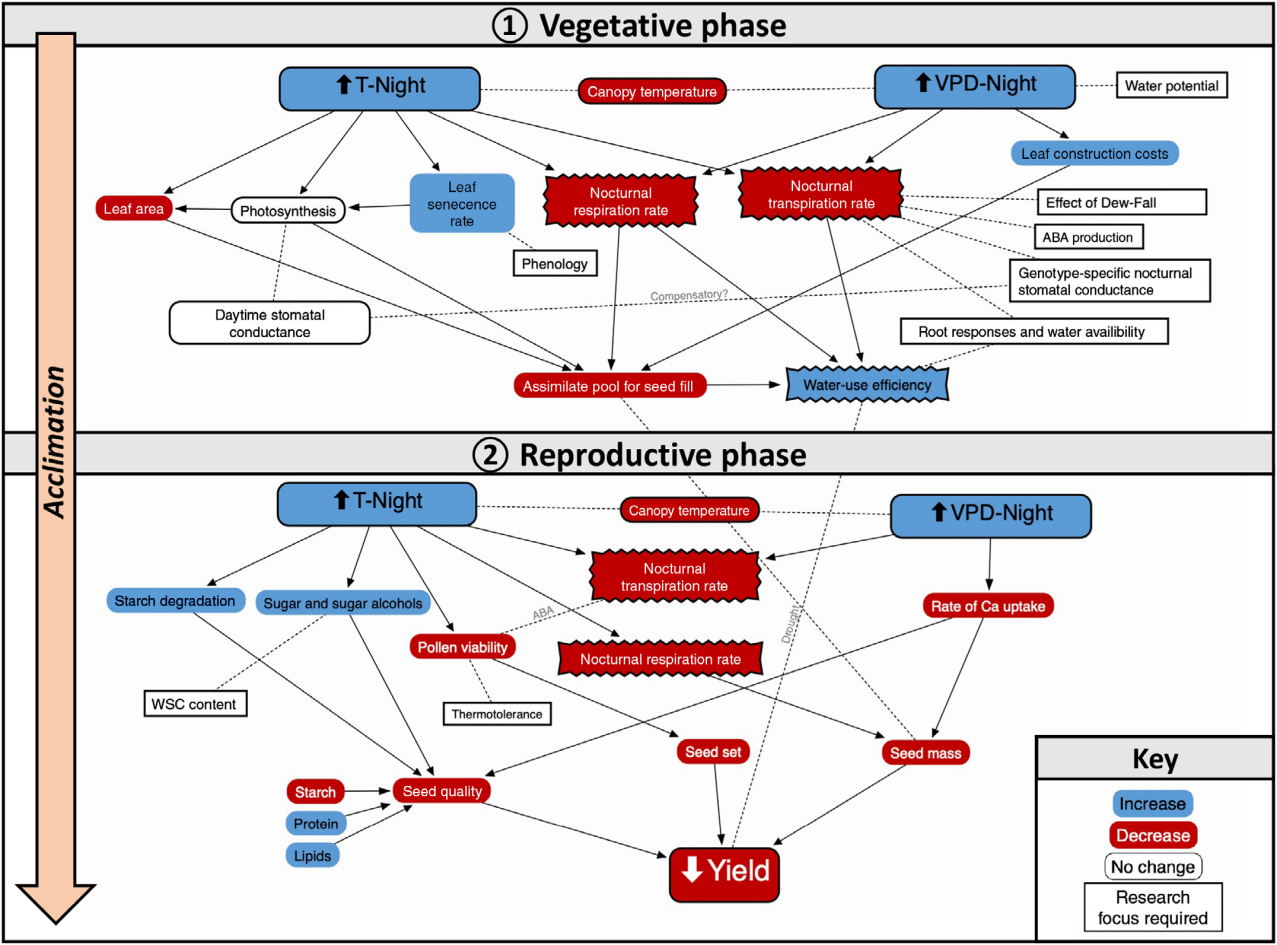
Plant processes acting at the leaf (vegetative) and reproductive phenologies impacted by high nocturnal temperatures (T‐Night) and subsequent increases in vapour pressure deficit (VPD) in the field. Adapted from Sadok & Jagadish (2020). Acclimation to heat is highlighted as a process affecting both the vegetative and reproductive stages in wheat. While the processes in ovals represent increases (blue) or decreases (red) in environmental conditions or plant traits, the jagged boxes highlight discrepancies highlighted in this study. These are connected by solid arrows, indicating specific impact, and dotted arrows, indicating potential impact. Finally, square‐edged boxes indicate areas where research efforts need to be focussed. WSC, water‐soluble carbohydrate.

Significant differences were determined between treatments for grain yield (GY), harvest index (HI) and biomass (BM; Fig. 6a,c,d) across combined year data. GY, BM, grain number (GN)N and crop growth rate (CGR) overall were significantly lower (*P* < 0.05) in the nocturnal heat treatment (Figs 6a,d, S12, S13). While GY varied between years, while BM was consistently lower across all 3 yr. On average, GY and BM were 3.83 and 5.75% lower, respectively, in the heat treatment relative to the control plots. HI was significantly higher under nocturnal heat conditions (+2.66%) relative to the control. However, some key yield component traits, such as TGW, were not significantly impacted by the heat treatment (Fig. 6b).

A significant year effect was seen for all evaluated yield and yield component parameters; however, interactions were not detected between year and genotype for GY, TGW, GN, SN, GPS, GWPS, GFP and GYPR. Interactions were found between year and treatment for GY, TGW, HI, PH, GWPS, GFR and GYPR. No differences were determined between years for biomass, grains per spike (GPS) and crop growth rate (Fig. S13). Finally, the only significant interaction found between year, treatment and genotype was for SN (Fig. S12).

## Discussion

There is growing evidence that crop responses to rising nocturnal temperatures are a key, underexplored source of variation in determining climate resilience. Although seemingly less severe, nocturnal warming can last much longer than excessive daytime heat, exposing the crop to a chronic rather than acute stress (Cox *et al*., 2020; Sadok & Jagadish, 2020). Often overlooked as a period of plant inertia, night‐time processes such as *g*_sn_, nocturnal transpiration, respiration, fluctuating carbohydrate reserves, hydraulic redistribution and water potential are part of an adaptive and integral diurnal strategy to enable survival to both short‐ and long‐term temperature stresses.

While traditional assessment of wheat heat tolerance, using techniques such as late‐season sowing (Langridge & Reynolds, 2021), may offer a broad determination of yield under high temperatures, this study highlights that genotypes classified as traditionally heat tolerant were sensitive to small increases in night‐time temperature even when no daytime stress was applied (Fig. 6). Furthermore, while nocturnal processes (*g*_sn_, *R*_d_, Ψ – Figs 3, S3, S7) responded to warmer nights, daytime measurements (*A*, *g*_s_ – Figs S4–S6) were significantly less sensitive suggesting distinct night/day metabolic acclimation.

Over the 3 yr of treatments, and under well‐irrigated conditions and artificially warmer nights, there was a cumulative negative effect on field grain yield; 3.83% overall – 1.9% per 1°C. At the leaf level, *g*_sn_ was genotype‐specific and more negatively affected by age than warming night‐time temperatures. As the plants aged, irrespective of treatment, rates of *g*_sn_ made up a greater proportion of total stomatal conductance as g_s_ declined more than *g*_sn_ (Table 2). Water potential mirrored the response of *g*_s_; increasing with age and in response to nocturnal heat (Fig. 2). Instead of an expected increase, *R*_d_ declined with age and in response to heat, indicative of rapid and sustained acclimation. As expected, late‐sown plants experienced much more severe declines in *A*, *g*_s_ and *g*_sn_, with *R*_d_ increasing (Fig. S11).

Using these responses and observations, we can contribute to the schematic proposed by Sadok & Jagadish (2020), drawing attention to key discrepancies in the response of wheat to nocturnal warming, while also highlighting key areas for research focus (Fig. 7).

### Stomatal responses and water availability

One of the core climactic changes associated with nocturnal warming is the decrease in available water, caused by increases in evapotranspiration, removing topsoil moisture and increasing canopy VPD. Using the T‐FACE set‐up, we observed minimal changes in canopy VPD with a 2°C increase in *T*_min_ (Fig. S2) suggesting *g*_sn_ (or *g*_s_) was not responding to changes in humidity. The increase in *g*_sn_ with age and the decline in leaf water potential in the heated plots predawn suggests a response to declining soil water availability. Less soil water uptake and higher *g*_sn_ could indicate attempts to improve nocturnal redistribution in the warmer plots as the season progressed (Huang *et al*., 1991; Lombardozzi *et al*., 2017).

Stomatal responses measured in the field are strongly linked to water availability with the decline in water potential greatest in the heated plots predawn (Fig. 2). Increases in *g*_sn_ have been linked to possible decreases in soil water availability, reducing available water at the roots produced by nocturnal hydraulic redistribution. A decrease in available topsoil water may be the indirect cause of genotype‐specific differences in *g*_sn_ (Saradadevi *et al*., 2015), with nocturnal heating having a greater detrimental impact on genotypes with wider, shallower rooting profiles and reducing the water available from heavy dewfall under control conditions. When compared to flood irrigation, drip‐irrigated wheat produces a greater number of longer roots at the surface (Chen *et al*., 2021). The decrease in water potential both predawn and midday in the heated treatments (Fig. 2) suggests a decline in water availability in the nocturnally heated plots. While changes in daytime *g*_s_ were minimal (Fig. S6), significant declines in *g*_sn_ (Figs 3, S8) suggest a greater sensitivity to water availability than daytime *g*_s_ (Gowing *et al*., 1990; Comstock, 2002). The lack of change in *g*_s_ also indicates changes in *g*_sn_ are not the result of the presence of the hormone, abscisic acid (ABA) which would likely affect daytime magnitudes of water loss both during the day (Fricker & Wilmer, 1996) and at night (Rawson & Clarke, 1988; Howard & Donovan, 2007). Finally, lower *g*_sn_ mirrored lower or indifferent leaf respiration rates (Fig. S7) and potential decrease in O_2_ consumption, which can occur with warming nights (Coast *et al*., 2021; Posch *et al*., 2022a,b).

Unlike growth room or glasshouse studies, the magnitude of *g*_sn_ and *g*_s_ appear uncoupled in the field (Fig. S10) and in response to heat (Fig. S11). Similar to previous studies (McAusland *et al*., 2021), genotype‐specific significant differences were determined in *g*_sn_ in the field (Fig. 3). The highest *g*_sn_ genotype, CIRNO, achieved similar values to those reported in growth room conditions, making up *c*. 9–19% of daytime rates of *g*_s_. *g*_sn_ declined with both age and nocturnal heat; however, the reduction in daytime *g*_s_ was more strongly influenced by age than nocturnal heating, enabling *g*_sn_ to make up a greater proportion of water loss as the plants matured (Table 3). It was also interesting to note that the magnitude of *g*_*sn*_ was leaf‐surface‐specific with the top of the leaf losing more water than the bottom at night (Table 2). This observation is supported by McAusland *et al*. (2021) and Wall *et al*. (2022), suggesting that the leaf surfaces can respond in both an independent manner but also coordinate to specific environmental stimulus (Wall *et al*., 2022). While Wall *et al*. focussed on responses to light, *g*_sn_ could be responding to other stimuli such as [CO_2_] or [O_2_] as a result of *R*_d_. For this study, measurements were taken 30 min–1 h after sunset; it is possible greater variation of *g*_sn_ exists throughout the night and predawn (Resco de dios *et al*., 2016), although dewfall in the field prevented these measurements being collected. More work is needed to link magnitudes of surface‐specific *g*_s_ and *g*_sn_ to root water availability, uptake and transport under heat (Wang *et al*., 2021).

### The role of respiration and acclimation in nocturnal heating

Rates of respiration are a major determinant in biomass accumulation, with up to 50% of carbon assimilated during the day being respired (Poorter *et al*., 1990). Typically, *R*_d_ has been shown to increase in response to short periods of nocturnal warming (e.g. Impa *et al*., 2019); however, our results show that respiration was genotype‐specific and declined in response to prolonged nocturnal heat (Fig. S7). In general, *R*_d_ increases in response to increasing temperature (Atkin & Tjoelker, 2003), supporting increased maintenance and potentially diverting carbon away from growth. Our data suggest the genotypes acclimated to the prolonged nocturnal warning and declining with age. Recent work by Posch *et al*. (2022a,b) provides strong evidence that nocturnal warming is a stronger driver of respiratory acclimation than daytime maximum temperatures. While the authors noted no change in CO_2_ release, oxygen consumption declined suggesting an increase in the respiratory quotient (Posch *et al*., 2022a,b). Interestingly, work by Bruhn *et al*. (2022) suggests that temperature accounts for less than half (48%) of the variation observed in *R*_d_ and that *R*_d_ declines through the night. While genotype‐specific differences in *R*_d_ were only observed at booting in 2020, assessment at different times of night and determining the rate of acclimation, rather than measurements of *R*_d_ at set phenological stages, may yield greater genetic variation between the genotypes (Bruhn *et al*., 2022).

In this study, *R*_d_ did increase in response to the severe heat stress associated with late sowing; interestingly, the average *T*_min_ of the regular‐sown, nocturnally heated plots (11.7°C) was not dissimilar to the average *T*_min_ of the late‐sown plots (10.3°C). This suggests the higher daytime temperatures (and potentially light intensities) elicited a greater negative impact on *R*_d_ than nocturnal temperatures alone.

Under high daytime temperatures, increases in *A* can support higher daytime respiration. However, at night, respiration relies on stored assimilates. As observed for booting plants in 2020, this can result in increases in *A* as a compensatory response to meet increased carbon demand (Turnbull *et al*., 2002; Fig. S5). There was a decline in leaf carbon content (Fig. 5a) for the regular‐sown plants across the 3 yr of measurements, suggesting a negative impact of nocturnal heating on the carbon supply; however, in two (2020 and 2022) of the three measurement years, the percentage of water‐soluble carbohydrates increased with nocturnal heating. Although not reflected in the gas exchange data, this suggests greater reserves of carbon at the stems and leaf sheaths to compensate for reduced photosynthates generated at the leaves.

### Nocturnal heating reduced grain yield and biomass production

Previous T‐FACE experiments have shown varied results relating to yield. For example, grain yield did not change significantly under combined, elevated day and night ambient temperature conditions in field‐grown rice (Wang *et al*., 2022). In winter bread wheat and durum wheat (CIRNO), increasing ambient temperatures by 1.5°C and 2°C throughout the entire growing season reduced grain yield by 16.3–19.6% and 33%, respectively (Tian *et al*., 2014; Garatuza‐Payan *et al*., 2018). A recent winter wheat study found that an increased nocturnal temperature by *c*. 1.4°C led to higher yields albeit in a single genotype (Fan *et al*., 2022).

Over 3 yr of measurements, we found that increasing nocturnal ambient temperature by *c*. 2°C significantly reduced yield and biomass production by 1.9% per 1°C increase in *T*_min_ despite average *T*_min_ being higher than the reported 14°C for decreasing yields in the literature (Fig. 1, Lobell & Ortiz‐Monasterio, 2007). Overall, this was supported by decreases in leaf C : N ratio implying a reduced carbon availability, altered partitioning or increased N assimilation/mobilisation. Interestingly, water‐soluble carbohydrates in the stems increased in the nocturnally heated plots at heading for 2020 and 2022 (Fig. 4), suggesting enhanced partition toward stem (Blum & Johnson, 1993); however, both control and heated WSC declined at maturity as the grain ripened to compensate for decreased photosynthetic contribution from the leaves due to age. Overall, there was no change in grain weight with increased nocturnal warming, suggesting the plants did not heavily rely on stored stem WSC to maintain yields under high nocturnal temperatures under irrigation (Zhang *et al*., 2014; Ovenden *et al*., 2017).

The response of harvest characteristics to nocturnal heat was genotype‐specific, with some demonstrating increases in grain yield and yield component traits under elevated nocturnal temperature (Fig. 6). Despite decreases in overall GY and biomass production, HI significantly increased overall in the heat treatment relative to the control, perhaps the result of larger reductions of biomass relative to grain yield under heat conditions. Despite observing significant changes to overall yield and biomass, 1000‐grain weight did not respond to the treatment. While nocturnal warming of the postanthesis has been implicated in reductions in grain weight (García *et al*., 2016), these data suggest that our reductions in yield are not due to changes in grain weight, but a decrease in the number of grains (Figs 6a,b, S12; Wang *et al*., 2020). Grain number (GN) is determined by duration of spike growth and fruiting efficiency or fertility index (Zhang *et al*., 2019; Langridge & Reynolds, 2021). In this study, nocturnal warming of *c*. 2°C was insufficient to significantly change the phenology of the plants under treatment (Table S2) suggesting a reduction in GN may be the result of reduced carbohydrate availability during spike development or spike fertility (Prasad *et al*., 2011; Draeger & Moore, 2017; Rieu *et al*., 2017; Narayanan *et al*., 2018).

Interestingly, genotypes that had high tolerance to heat were not always the highest yielding (and vice versa). For example, GID‐2465 and GID‐7129721 were classified as having high heat tolerance. However, both were among the lowest yielding genotypes under warmer nights. Previous heat tolerance was determined via exposure to long‐term heat stress through late sowing where plants would be exposed to higher day‐ and night‐time temperatures throughout development. However, the relative increase in daytime temperature far exceeds the increase in night‐time temperature. Although less severe by magnitude, nocturnal warming can last much longer than excessive daytime heat (Sillmann *et al*., 2013; Sadok & Jagadish, 2020), exposing the crop to a chronic rather than acute stress.

These results suggest that evaluation of heat performance largely based on *T*_max_, which is experienced during the day, may not help inform performance under elevated *T*_min_. Consequently, current high‐temperature‐tolerant genotypes may not be adapted to rising *T*_min_, requiring a redefinition of metrics for heat tolerance A greater understanding of the mechanism of yield – driven by *T*_min_, *T*_max_ and their interactions is needed. Future emphasis could be placed on understanding the mechanisms of individual wheat genotypes that had increases or no significant penalty to yield and yield components, such as GID‐7171118 (Fig. 6). For example, processes such as the flexibility of PSII (Coast *et al*., 2022), the magnitude and ability of respiration to acclimate (Posch *et al*., 2019), pollen temperature sensitivity and the degree of acquired thermotolerance from past increases in temperature (Wang *et al*., 2012; Djanaguiraman *et al*., 2013; Echer *et al*., 2014; Müller & Rieu, 2016) may all contribute to genotype‐specific responses to rising *T*_min_.

### Conclusions and future considerations

There is growing awareness of the negative impact rising nocturnal temperatures has on crop yields; however, understanding the physiological responses to this phenomenon remains limited. In Fig. 7, we highlight how this study fits within our current understanding of nocturnal temperature stress. Genotype‐specific differences in day‐ vs night‐time heat tolerance strongly suggest assessment of nocturnal thermotolerance should be considered when selecting ‘heat tolerant’ genotypes for future climates. We also acknowledge that high temperatures impact grain quality (Hein *et al*., 2022; Impa *et al*., 2019) as well as quantity.

While there is some evidence that nocturnal warming at key growth stages and under irrigated conditions could improve yields (Chen *et al*., 2014; Fan *et al*., 2022), it is more likely that a combination of stresses, accelerated by warmer nights, contributes to their decline; for example, decreased water availability, high VPD and combined high *T*_max_. There is a lack of fieldwork investigating the combined impact of these nocturnal factors on leaf‐level traits and how they interact to ultimately determine yield. Nocturnal conductance remains a genotype‐specific, elusive night‐time trait which plays a role in whole‐plant water efficiency, while very little work has connected *g*_sn_ with root development and physiology under nocturnal heat, or the potential interactions with night‐specific field traits such as dewfall – an important characteristic of nocturnal foliar uptake in trees but not commonly observed in crop species (Schreel & Steppe, 2020). With this in mind, we suggest future work should prioritise elucidating the variation in nocturnal heat tolerance and determination of the impact of diurnal water acquisition on the magnitude of *g*_sn_.

## Competing interests

None declared.

## Author contributions

EY, JGP, EHM, MR, GM, LM and FP conceived the original research plan. EY, JGP, EHM, MR, GM, LM, FP and SP designed the field experiment. LM, GM, LGA‐S, SP, FP, EY and JGP performed the field experiments, collected and analysed the data. LM, LGA‐S, SP and FP wrote the manuscript with contributions from co‐authors. EHM, MR, EY and JGP obtained project funding. All authors contributed to the article and approved the submitted version. LM and LGA‐S contributed equally to this work.

## Supporting information


**Fig. S1** Photograph of the infrared heating set‐up and arrangement of the field plots.
**Fig. S2** Vapour pressure deficit measured across all 3 yr in the regular‐sown control.
**Fig. S3** Response of leaf water potential predawn and at midday between 2020 and 2022.
**Fig. S4** Response of *g*_s_ and *g*_sn_ between 2020 and 2022 as measured using the porometer.
**Fig. S5** Response of photosynthetic assimilation to nocturnal heating.
**Fig. S6** Response of daytime stomatal conductance to nocturnal heating.
**Fig. S7** Response of nocturnal respiration to nocturnal heating.
**Fig. S8** Response of nocturnal stomatal conductance to nocturnal heating.
**Fig. S9** Comparing the responses of *g*_s_ and *g*_sn_ under control and nocturnally heated conditions.
**Fig. S10** Correlating *g*_s_ and *g*_sn_ at different growth stages and in response to nocturnal heat.
**Fig. S11** Comparing the control and nocturnally heated responses of *A*, *g*_s_, *R*_d_ and *g*_sn_ to measurements made on late‐sown plants.
**Fig. S12** Distribution of grain number, spike number, grain per spike and grain weight per spike.
**Fig. S13** Distribution of plant height, grain‐filling period, grain yield production rate and crop growth rate.
**Table S1** Settings used for the porometer measurements during the day and at night.
**Table S2** Efficiency of the heating system.
**Table S3** Meteorological data of the heat and control plots across the whole crop.
**Table S4** Days to booting, heading and maturity across years and treatments season by Stage × Time × Year.
Please note: Wiley is not responsible for the content or functionality of any Supporting Information supplied by the authors. Any queries (other than missing material) should be directed to the *New Phytologist* Central Office.

## Data Availability

All data supporting the findings of this study are accessible and published online at www.figshare.com under the DOI: 10.6084/m9.figshare.23104817 and can be accessed using the following link; https://figshare.com/s/6a469e2f6258bfcc27df.

## References

[nph19075-bib-0101] dos Anjos L , Pandey PK , Moraes TA , Feil R , Lunn JE , Stitt M . 2018. Feedback regulation by trehalose 6‐phosphate slows down starch mobilization below the rate that would exhaust starch reserves at dawn in Arabidopsis leaves. Plant Direct 2: e00078. 31245743 10.1002/pld3.78 PMC6508811

[nph19075-bib-0102] Argentel‐Martínez L , Garatuza‐Payan J , Yepez EA , Arredondo T , de Los Santos‐Villalobos S . 2019. Water regime and osmotic adjustment under warming conditions on wheat in the Yaqui Valley, Mexico. PeerJ 12: e7029. 10.7717/peerj.7029 PMC6570998 31223527

[nph19075-bib-0001] Atkin OK , Tjoelker MG . 2003. Thermal acclimation and the dynamic response of plant respiration to temperature. Trends in Plant Science 8: 343–351. 12878019 10.1016/S1360-1385(03)00136-5

[nph19075-bib-0002] Bahuguna RN , Solis CA , Shi W , Jagadish KSV . 2017. Post‐flowering night respiration and altered sink activity account for high night temperature‐induced grain yield and quality loss in rice (*Oryza sativa* L.). Physiologia Plantarum 159: 59–73. 27513992 10.1111/ppl.12485

[nph19075-bib-0103] Bheemanahalli R , Knight M , Quinones C , Doherty CJ , Jagadish SK . 2021. Genome‐wide association study and gene network analyses reveal potential candidate genes for high night temperature tolerance in rice. Scientific Reports 11: 6747. 33762605 10.1038/s41598-021-85921-z PMC7991035

[nph19075-bib-0003] Blum A , Johnson JW . 1993. Wheat cultivars respond differently to a drying top soil and a possible non‐hydraulic root signal. Journal of Experimental Botany 44: 1149–1153.

[nph19075-bib-0004] Bruhn D , Newman F , Hancock M , Povlsen P , Slot M , Sitch S , Drake J , Weedon GP , Clark DB , Pagter M *et al*. 2022. Nocturnal plant respiration is under strong non‐temperature control. Nature Communications 13: 1–10. 10.1038/s41467-022-33370-1 PMC9512894 36163192

[nph19075-bib-0104] Caird MA , Richards JH , Donovan LA . 2007. Nighttime stomatal conductance and transpiration in C3 and C4 plants. Plant Physiology 143: 4–10. 17210908 10.1104/pp.106.092940 PMC1761996

[nph19075-bib-0006] Chen J , Tian Y , Zhang X , Zheng C , Song Z , Deng A , Zhang W . 2014. Nighttime warming will increase winter wheat yield through improving plant development and grain growth in North China. Journal of Plant Growth Regulation 33: 397–407.

[nph19075-bib-0007] Chen R , Xiong XP , Cheng WH . 2021. Root characteristics of spring wheat under drip irrigation and their relationship with aboveground biomass and yield. Scientific Reports 11: 1–14. 33649480 10.1038/s41598-021-84208-7 PMC7921688

[nph19075-bib-0105] Coast O , Ellis RH , Murdoch AJ , Quiñones C , Jagadish KS . 2014. High night temperature induces contrasting responses for spikelet fertility, spikelet tissue temperature, flowering characteristics and grain quality in rice. Functional Plant Biology 42: 149–161. 10.1071/FP14104 32480661

[nph19075-bib-0008] Coast O , Posch BC , Bramley H , Gaju O , Richards RA , Lu M , Ruan YL , Trethowan R , Atkin OK . 2021. Acclimation of leaf photosynthesis and respiration to warming in field‐grown wheat. Plant, Cell & Environment 44: 2331–2346. 10.1111/pce.13971 33283881

[nph19075-bib-0009] Coast O , Posch BC , Rognoni BG , Bramley H , Gaju O , Mackenzie J , Pickles C , Kelly AM , Lu M , Ruan YL *et al*. 2022. Wheat photosystem II heat tolerance: evidence for genotype‐by‐environment interactions. The Plant Journal 111: 1368–1382. 35781899 10.1111/tpj.15894

[nph19075-bib-0010] Comstock JP . 2002. Hydraulic and chemical signalling in the control of stomatal conductance and transpiration. Journal of Experimental Botany 53: 195–200. 11807122 10.1093/jexbot/53.367.195

[nph19075-bib-0011] Cox DT , Maclean IM , Gardner AS , Gaston KJ . 2020. Global variation in diurnal asymmetry in temperature, cloud cover, specific humidity and precipitation and its association with leaf area index. Global Change Biology 26: 7099–7111. 32998181 10.1111/gcb.15336

[nph19075-bib-0106] Daley MJ , Phillips NG . 2006. Interspecific variation in nighttime transpiration and stomatal conductance in a mixed New England deciduous forest. Tree Physiology 24: 411–419. 10.1093/treephys/26.4.411 16414920

[nph19075-bib-0107] Davy R , Esau I , Chernokulsky A , Outten S , Zilitinkevich S . 2017. Diurnal asymmetry to the observed global warming. International Journal of Climatology 37: 79–93.

[nph19075-bib-0013] Djanaguiraman M , Prasad PVV , Schapaugh WT . 2013. High day‐ or nighttime temperature alters leaf assimilation, reproductive success, and phosphatidic acid of pollen grain in soybean [*Glycine max* (L.) Merr.]. Crop Science 53: 1594–1604.

[nph19075-bib-0014] Draeger T , Moore G . 2017. Short periods of high temperature during meiosis prevent normal meiotic progression and reduce grain number in hexaploid wheat (*Triticum aestivum* L.). Theoretical and Applied Genetics 130: 1785–1800. 28550436 10.1007/s00122-017-2925-1 PMC5565671

[nph19075-bib-0015] Echer FR , Oosterhuis DM , Loka DA , Rosolem CA . 2014. High night temperatures during the floral bud stage increase the abscission of reproductive structures in cotton. Journal of Agronomy and Crop Science 200: 191–198.

[nph19075-bib-0108] Even M , Sabo M , Meng D , Kreszies T , Schreiber L , Fricke W . 2018. Night‐time transpiration in barley (*Hordeum vulgare*) facilitates respiratory carbon dioxide release and is regulated during salt stress. Annals of Botany 122: 569–582. 29850772 10.1093/aob/mcy084 PMC6153476

[nph19075-bib-0016] Fan Y , Lv Z , Qin B , Yang J , Ren K , Liu Q , Jiang F , Zhang W , Ma S , Ma C *et al*. 2022. Night warming at the vegetative stage improves pre‐anthesis photosynthesis and plant productivity involved in grain yield of winter wheat. Plant Physiology and Biochemistry 186: 19–30. 35797916 10.1016/j.plaphy.2022.06.015

[nph19075-bib-0017] Fischer RA . 1985. Number of kernels in wheat crops and the influence of solar radiation and temperature. The Journal of Agricultural Science 105: 447–461.

[nph19075-bib-0109] Fricke W . 2019. Night‐time transpiration – favouring growth? Trends in Plant Science 24: 311–317. 30770287 10.1016/j.tplants.2019.01.007

[nph19075-bib-0018] Fricker MD , Wilmer C . 1996. Stomata, 2^nd^ edn. London, UK: Chapman & Hall.

[nph19075-bib-0019] Garatuza‐Payan J , Argentel‐Martinez L , Yepez EA , Arredondo T . 2018. Initial response of phenology and yield components of wheat (*Triticum durum* L., CIRNO C2008) under experimental warming field conditions in the Yaqui Valley. PeerJ 6: e5064. 29942702 10.7717/peerj.5064 PMC6015750

[nph19075-bib-0110] García GA , Dreccer MF , Miralles DJ , Serrago RA . 2015. High night temperatures during grain number determination reduce wheat and barley grain yield: a field study. Global Change Biology 21: 4153–4164. 26111197 10.1111/gcb.13009

[nph19075-bib-0020] García GA , Serrago RA , Dreccer MF , Miralles DJ . 2016. Post‐anthesis warm nights reduce grain weight in field‐grown wheat and barley. Field Crops Research 195: 50–59.

[nph19075-bib-0021] Gowing DJ , Davies WJ , Jones HG . 1990. A positive root‐sourced signal as an indicator of soil drying in apple, Malus × domestica Borkh. Journal of Experimental Botany 41: 1535–1540.

[nph19075-bib-0022] Harte J , Shaw R . 1995. Shifting dominance within a montane vegetation community: results of a climate‐warming experiment. Science 267: 876–880. 17813919 10.1126/science.267.5199.876

[nph19075-bib-2000] Hein NT , Impa SM , Wagner D , Bheemanahalli R , Kumar R , Tiwari M , Prasad PV , Tilley M , Wu X , Neilsen M *et al*. 2022. Grain micronutrient composition and yield components in field-grown wheat are negatively impacted by high night-time temperature. Cereal Chemistry 99: 615–624.

[nph19075-bib-0023] Howard AR , Donovan LA . 2007. Helianthus nighttime conductance and transpiration respond to soil water but not nutrient availability. Plant Physiology 143: 145–155. 17142487 10.1104/pp.106.089383 PMC1761982

[nph19075-bib-0024] Huang BR , Taylor HM , McMichael BL . 1991. Effects of temperature on the development of metaxylem in primary wheat roots and its hydraulic consequence. Annals of Botany 67: 163–166.

[nph19075-bib-0025] Impa SM , Sunoj VSJ , Krassovskaya I , Bheemanahalli R , Obata T , Jagadish SVK . 2019. Carbon balance and source‐sink metabolic changes in winter wheat exposed to high night‐time temperature. Plant, Cell & Environment 42: 1233–1246. 10.1111/pce.13488 30471235

[nph19075-bib-0111] Kimball BA . 2005. Theory and performance of an infrared heater for ecosystem warming. Global Change Biology 11: 2041–2056.

[nph19075-bib-0027] Kimball BA . 2015. Using canopy resistance for infrared heater control when warming open‐field plots. Agronomy Journal 107: 1105–1112.

[nph19075-bib-0028] Kimball BA , Conley MM , Wang S , Lin X , Luo C , Morgan J , Smith D . 2008. Infrared heater arrays for warming ecosystem field plots. Global Change Biology 14: 309–320.

[nph19075-bib-0029] Kimball BA , White JW , Ottman MJ , Wall GW , Bernacchi CJ , Morgan JA , Smith DP . 2015. Predicting canopy temperatures and infrared heater energy requirements for warming field plots. Agronomy Journal 107: 129–141.

[nph19075-bib-0030] Langridge P , Reynolds M . 2021. Breeding for drought and heat tolerance in wheat. Theoretical and Applied Genetics 134: 1753–1769. 33715017 10.1007/s00122-021-03795-1

[nph19075-bib-0112] Lesjak J , Calderini DF . 2017. Increased night temperature negatively affects grain yield, biomass and grain number in Chilean quinoa. Frontiers in Plant Science 8: 352. 28386266 10.3389/fpls.2017.00352 PMC5362734

[nph19075-bib-0113] Lin T , Okamoto Y , Nagasaki Y , Shiraiwa T . 2021. The influence of high night temperature on yield and physiological attributes of Soybean cv. Fukuyutaka. Plant Production Science 24: 267–278.

[nph19075-bib-0031] Lobell DB , Ortiz‐Monasterio JI . 2007. Impacts of day versus night temperatures on spring wheat yields: a comparison of empirical and CERES model predictions in three locations. Agronomy Journal 99: 469–477.

[nph19075-bib-0032] Lobell DB , Ortiz‐Monasterio JI , Asner GP , Matson PA , Naylor RL , Falcon WP . 2005. Analysis of wheat yield and climatic trends in Mexico. Field Crops Research 94: 250–256.

[nph19075-bib-0114] Loka DA , Oosterhuis DM . 2010. Effect of high night temperatures on cotton respiration, ATP levels and carbohydrate content. Environmental and Experimental Botany 68: 258–263.

[nph19075-bib-0033] Lombardozzi DL , Zeppel MJB , Fisher RA , Tawfik A . 2017. Representing nighttime and minimum conductance in CLM4. 5: global hydrology and carbon sensitivity analysis using observational constraints. Geoscientific Model Development 10: 321–331.

[nph19075-bib-0034] McAusland L , Smith KE , Williams A , Molero G , Murchie EH . 2021. Nocturnal stomatal conductance in wheat is growth‐stage specific and shows genotypic variation. New Phytologist 232: 162–175. 34143507 10.1111/nph.17563

[nph19075-bib-0037] Moore CE , Meacham‐Hensold K , Lemonnier P , Slattery RA , Benjamin C , Bernacchi CJ , Lawson T , Cavanagh AP . 2021. The effect of increasing temperature on crop photosynthesis: from enzymes to ecosystems. Journal of Experimental Botany 72: 2822–2844. 33619527 10.1093/jxb/erab090 PMC8023210

[nph19075-bib-0038] Müller F , Rieu I . 2016. Acclimation to high temperature during pollen development. Plant Reproduction 29: 107–118. 27067439 10.1007/s00497-016-0282-x PMC4909792

[nph19075-bib-0039] Narayanan S , Prasad PVV , Fritz AK , Boyle DL , Gill BS . 2015. Impact of high night‐time and high daytime temperature stress on winter wheat. Journal of Agronomy and Crop Science 201: 206–218.

[nph19075-bib-0040] Narayanan S , Prasad PVV , Welti R . 2018. Alterations in wheat pollen lipidome during high day and night temperature stress. Plant, Cell & Environment 41: 1749–1761. 10.1111/pce.13156 PMC6713575 29377219

[nph19075-bib-0041] Nijs I , Kockelbergh F , Teughels H , Blum H , Hendrey G , Impens I . 1996. Free air temperature increase (FATI): a new tool to study global warming effects on plants in the field. Plant, Cell & Environment 19: 495–502.

[nph19075-bib-0042] Ovenden B , Milgate A , Lisle C , Wade LJ , Rebetzke GJ , Holland JB . 2017. Selection for water‐soluble carbohydrate accumulation and investigation of genetic × environment interactions in an elite wheat breeding population. Theoretical and Applied Genetics 130: 2445–2461. 28852799 10.1007/s00122-017-2969-2

[nph19075-bib-0115] Pask AJD , Pietragalla Mullan JD , Reynolds MP , eds. 2012. Physiological breeding II: a field guide to wheat phenotyping. Toluca, Mexico: CIMMYT.

[nph19075-bib-0116] Peng S , Huang J , Sheehy JE , Laza RC , Visperas RM , Zhong X , Centeno GS , Khush GS , Cassman KG . 2004. Rice yields decline with higher night temperature from global warming. Proceedings of the National Academy of Sciences, USA 101: 9971–9975. 10.1073/pnas.0403720101 PMC454199 15226500

[nph19075-bib-0043] Pietragalla J , Mullan DM , Reynolds MP . 2012. Physiological breeding II: a field guide to wheat phenotyping. Ciudad Obregon, Mexico: CIMMYT.

[nph19075-bib-0044] Poorter H , Remkes C , Lambers H . 1990. Carbon and nitrogen economy of 24 wild species differing in relative growth rate. Plant Physiology 94: 621–627. 16667757 10.1104/pp.94.2.621 PMC1077277

[nph19075-bib-0045] Posch BC , Hammer J , Atkin OK , Bramley H , Ruan Y‐L , Trethowan R , Coast O . 2022a. Wheat photosystem II heat tolerance responds dynamically to short‐ and long‐term warming. Journal of Experimental Botany 73: 3268–3282. 35604885 10.1093/jxb/erac039 PMC9127437

[nph19075-bib-0046] Posch BC , Kariyawasam BC , Bramley H , Coast O , Richards RA , Reynolds MP , Trethowan R , Atkin OK . 2019. Exploring high temperature responses of photosynthesis and respiration to improve heat tolerance in wheat. Journal of Experimental Botany 70: 5051–5069. 31145793 10.1093/jxb/erz257

[nph19075-bib-0047] Posch BC , Zhai D , Coast O , Scafaro AP , Bramley H , Reich P , Ruan YL , Trethowan R , Way DA , Atkin O . 2022b. Wheat respiratory O_2_ consumption falls with night warming alongside greater respiratory CO_2_ loss and reduced biomass. Journal of Experimental Botany 73: 915–926. 34652413 10.1093/jxb/erab454

[nph19075-bib-0048] Prasad PVV , Djanaguiraman M , Prasad PVV , Djanaguiraman M . 2011. High night temperature decreases leaf photosynthesis and pollen function in grain sorghum. Functional Plant Biology 38: 993–1003. 32480957 10.1071/FP11035

[nph19075-bib-0117] R Core Team . 2016. R: a language and environment for statistical computing. R v.4.2.1 (2022‐06‐23) – “funny‐looking kid”. Vienna, Austria: R Foundation for Statistical Computing.

[nph19075-bib-0049] Rawson HM , Clarke JM . 1988. Nocturnal transpiration in wheat. Functional Plant Biology 15: 397–406.

[nph19075-bib-0050] Resco de Dios V , Loik ME , Smith R , Aspinwall MJ , Tissue DT . 2016. Genetic variation in circadian regulation of nocturnal stomatal conductance enhances carbon assimilation and growth. Plant, Cell & Environment 39: 3–11. 10.1111/pce.12598 26147129

[nph19075-bib-0051] Rieu I , Twell D , Firon N . 2017. Pollen development at high temperature: from acclimation to collapse. Plant Physiology 173: 1967–1976. 28246296 10.1104/pp.16.01644 PMC5373052

[nph19075-bib-0052] Sadok W , Jagadish SVK . 2020. The hidden costs of nighttime warming on yields. Trends in Plant Science 25: 644–651. 32526169 10.1016/j.tplants.2020.02.003

[nph19075-bib-0053] Sadok W , Schoppach R . 2019. Potential involvement of root auxins in drought tolerance by modulating nocturnal and daytime water use in wheat. Annals of Botany 124: 969–978. 30918962 10.1093/aob/mcz023 PMC6881217

[nph19075-bib-0055] Saradadevi R , Bramley H , Palta JA , Edwards E , Siddique KHM , Saradadevi R , Bramley H , Palta JA , Edwards E , Siddique KHM . 2015. Root biomass in the upper layer of the soil profile is related to the stomatal response of wheat as the soil dries. Functional Plant Biology 43: 62–74. 32480442 10.1071/FP15216

[nph19075-bib-0118] Sayre KD , Rajaram S , Fischer RA . 1997. Yield potential progress in short bread wheats in northwest Mexico. Crop Science 37: 36–42.

[nph19075-bib-0056] Schreel JD , Steppe K . 2020. Foliar water uptake in trees: negligible or necessary? Trends in Plant Science 25: 590–603. 32407698 10.1016/j.tplants.2020.01.003

[nph19075-bib-0119] Shi W , Yin X , Struik PC , Xie F , Schmidt RC , Jagadish KS . 2016. Grain yield and quality responses of tropical hybrid rice to high night‐time temperature. Field Crops Research 190: 18–25.

[nph19075-bib-0057] Sillmann J , Kharin VV , Zwiers FW , Zhang X , Bronaugh D . 2013. Climate extremes indices in the CMIP5 multimodel ensemble: part 2. Future climate projections. Journal of Geophysical Research: Atmospheres 118: 2473–2493.

[nph19075-bib-0058] Tian Y , Zheng C , Chen J , Chen C , Deng A , Song Z , Zhang B , Zhang W . 2014. Climatic warming increases winter wheat yield but reduces grain nitrogen concentration in east China. PLoS ONE 9: e95108. 24736557 10.1371/journal.pone.0095108 PMC3988157

[nph19075-bib-0059] Turnbull MH , Murthy R , Griffin KL . 2002. The relative impacts of daytime and night‐time warming on photosynthetic capacity in Populus deltoides. Plant, Cell & Environment 25: 1729–1737.

[nph19075-bib-0061] Wall S , Vialet‐Chabrand S , Davey P , van Rie J , Galle A , Cockram J , Lawson T . 2022. Stomata on the abaxial and adaxial leaf surfaces contribute differently to leaf gas exchange and photosynthesis in wheat. New Phytologist 235: 1743–1756. 35586964 10.1111/nph.18257 PMC9545378

[nph19075-bib-0062] Wang H , Yang T , Chen J , Bell SM , Wu S , Jiang Y , Sun Y , Zeng Y , Zeng Y , Pan X *et al*. 2022. Effects of free‐air temperature increase on grain yield and greenhouse gas emissions in a double rice cropping system. Field Crops Research 281: 108489.

[nph19075-bib-0063] Wang X , Cai J , Liu F , Jin M , Yu H , Jiang D , Wollenweber B , Dai T , Cao W . 2012. Pre‐anthesis high temperature acclimation alleviates the negative effects of post‐anthesis heat stress on stem stored carbohydrates remobilization and grain starch accumulation in wheat. Journal of Cereal Science 55: 331–336.

[nph19075-bib-0064] Wang Y , Anderegg WRL , Venturas MD , Trugman AT , Yu K , Frankenberg C . 2021. Optimization theory explains nighttime stomatal responses. New Phytologist 230: 1550–1561. 33576001 10.1111/nph.17267

[nph19075-bib-0065] Wang Y , Tao H , Zhang P , Hou X , Sheng D , Tian B , Wang P , Huang S . 2020. Reduction in seed set upon exposure to high night temperature during flowering in maize. Physiologia Plantarum 169: 73–82. 31747055 10.1111/ppl.13049

[nph19075-bib-0120] Welch JR , Vincent JR , Auffhammer M , Moya PF , Dobermann A , Dawe D . 2010. Rice yields in tropical/subtropical Asia exhibit large but opposing sensitivities to minimum and maximum temperatures. Proceedings of the National Academy of Sciences, USA 107: 14562–14567. 10.1073/pnas.1001222107 PMC2930450 20696908

[nph19075-bib-0066] Yemm EW , Willis A . 1954. The estimation of carbohydrates in plant extracts by anthrone. Biochemical Journal 57: 508–514. 13181867 10.1042/bj0570508 PMC1269789

[nph19075-bib-0121] Zadoks JC , Chang TT , Konzak CF . 1974. A decimal code for the growth stages of cereals. Weed Research 14: 415–421.

[nph19075-bib-0067] Zhang B , Li W , Chang X , Li R , Jing R . 2014. Effects of favorable alleles for water‐soluble carbohydrates at grain filling on grain weight under drought and heat stresses in wheat. PLoS ONE 9: e102917. 25036550 10.1371/journal.pone.0102917 PMC4103880

[nph19075-bib-0068] Zhang H , Richards R , Riffkin P , Berger J , Christy B , O'Leary G , Acuna TB , Merry A . 2019. Wheat grain number and yield: The relative importance of physiological traits and source‐sink balance in southern Australia. European Journal of Agronomy 110: 125935.

