# Supplementary material for: Night‐time warming in the field reduces nocturnal stomatal conductance and grain yield but does not alter daytime physiological responses
Source: New Phytol. 2023 Jul 10;239(5):1622–36. doi: 10.1111/nph.19075 (PMC10952344; doi:10.1111/nph.19075)
Supplement: Supplementary file 1 — Fig. S1 Photograph of the infrared heating set‐up and arrangement of the field plots. Fig. S2 Vapour pressure deficit measured across all 3 yr in the regular‐sown control. Fig. S3 Response of leaf water potential predawn and at midday between 2020 and 2022. Fig. S4 Response of g s and g sn between 2020 and 2022 as measured using the porometer. Fig. S5 Response of photosynthetic assimilation to nocturnal heating. Fig. S6 Response of daytime stomatal conductance to nocturnal heating. Fig. S7 Response of nocturnal respiration to nocturnal heating. Fig. S8 Response of nocturnal stomatal conductance to nocturnal heating. Fig. S9 Comparing the responses of g s and g sn under control and nocturnally heated conditions. Fig. S10 Correlating g s and g sn at different growth stages and in response to nocturnal heat. Fig. S11 Comparing the control and nocturnally heated responses of A, g s, R d and g sn to measurements made on late‐sown plants. Fig. S12 Distribution of grain number, spike number, grain per spike and grain weight per spike. Fig. S13 Distribution of plant height, grain‐filling period, grain yield production rate and crop growth rate. Table S1 Settings used for the porometer measurements during the day and at night. Table S2 Efficiency of the heating system. Table S3 Meteorological data of the heat and control plots across the whole crop. Table S4 Days to booting, heading and maturity across years and treatments season by Stage × Time × Year. Please note: Wiley is not responsible for the content or functionality of any Supporting Information supplied by the authors. Any queries (other than missing material) should be directed to the New Phytologist Central Office. [file NPH-239-1622-s001.pdf]

New Phytologist Supporting Information

Article title:

Night-time warming in the field reduces nocturnal stomatal conductance and grain yield but does not alter daytime physiological responses

Authors:

Dr Lorna McAusland[2†]
Dr Liana G. Acevedo-Siaca[1†]
Dr R. Suzuky Pinto[3]
Dr Francisco Pinto[1]
Dr Gemma Molero[1]
Dr Jaime Garatuza-Payan[3]
Dr Matthew Reynolds[1]
Prof. Erik Murchie[2*]
Dr. Enrico A. Yepez[3]

Article acceptance date:

11th May 2023

List Of Supplementary Tables (4) and Figures (13)

Tables

Table S1 The settings used for the porometer measurements during the day and at night

Table S2 Efficiency of the heating system (%)

Table S3 Meteorological data of the heat and control plots across the whole crop

Table S4 Days to booting, heading, and maturity across years and treatments

season by Stage × Time × Year.

Figures

Figure S1 Photo of the Infra-Red heating set-up and arrangement of the field plots

Figure S2 Vapour pressure deficit measured across all three years in the regular sown control

Figure S3 Response of leaf water potential predawn and at midday between 2020-2022

Figure S4 Response of g_s and g_{sn} between 2020-2022 as measured using the porometer

Figure S5 The response of photosynthetic assimilation to nocturnal heating

Figure S6 The response of daytime stomatal conductance to nocturnal heating

Figure S7 The response of nocturnal respiration to nocturnal heating

Figure S8 The response of nocturnal stomatal conductance to nocturnal heating.

Figure S9 Comparing the responses of g_s and g_{sn} under control and nocturnally heated conditions

Figure S10 Correlating g_s and g_{sn} at different growth stages and in response to nocturnal heat

Figure S11 Comparing the control and nocturnally heated responses of A, g_s, R_d and g_{sn} to measurements made on late sown plants

Figure S12 Distribution of grain number, spike number, grain per spike, and grain weight per spike.

Figure S13 Distribution of plant height, grain filling period, grain yield production rate, and crop growth rate.

Table S1. Settings for the porometer (Li-600, Li-Cor Biosciences Ltd, Nebraska, USA) used at night (a) and during the day (b).

	(**a**) *Nocturnal Measurements*			
	**Parameter**	**Value**	**units**	**Notes**
	Protocol Name:	Autogsw+F_NIGHT	NA	
	gsw_stability	0.005 over 2s	(mol m^{-2} s^{-1}) s^{-1}	**Fast**
	F_stability	5 over 2s	s^{-1}	**Fast**
	Flow Rate	150	μmol s^{-1}	**High**
	Match Frequency	10	minutes	
	Dark adapted?	YES	NA	
	Flash type	Multiphase	NA	
	Flash intensity	6000	μmol m^{-2} s^{-1}	
	Flash length	800	ms	
	Leaf Abs	0.8	NA	
	Fraction Abs PSII	0.5	NA	
	Modulation Rate	500	Hz	
	Integrated Modulation Intensity	0.0667	μmol m^{-2} s^{-1}	
	(**b**) *Daytime Measurements*			
	**Parameter**	**Value**	**units**	**Notes**
	Protocol Name:	Auto gsw+F_DAY	NA	
	gsw_stability	0.001 over 2s	(mol m^{-2} s^{-1}) s^{-1}	**Medium**
	F_stability	5 over 2s	s^{-1}	**Fast**
	Flow Rate	150	μmol s^{-1}	**High**
	Match Frequency	10	minutes	
	Dark adapted?	NO	NA	
	Flash type	Multiphase	NA	
	Flash intensity	7000	μmol m^{-2} s^{-1}	
	Ramp Amount	25	%	
	Leaf Abs	0.8	NA	
	Fraction Abs PSII	0.5	NA	
	Modulation Rate	500	Hz	
	Integrated Modulation Intensity	6.67	μmol m^{-2} s^{-1}	

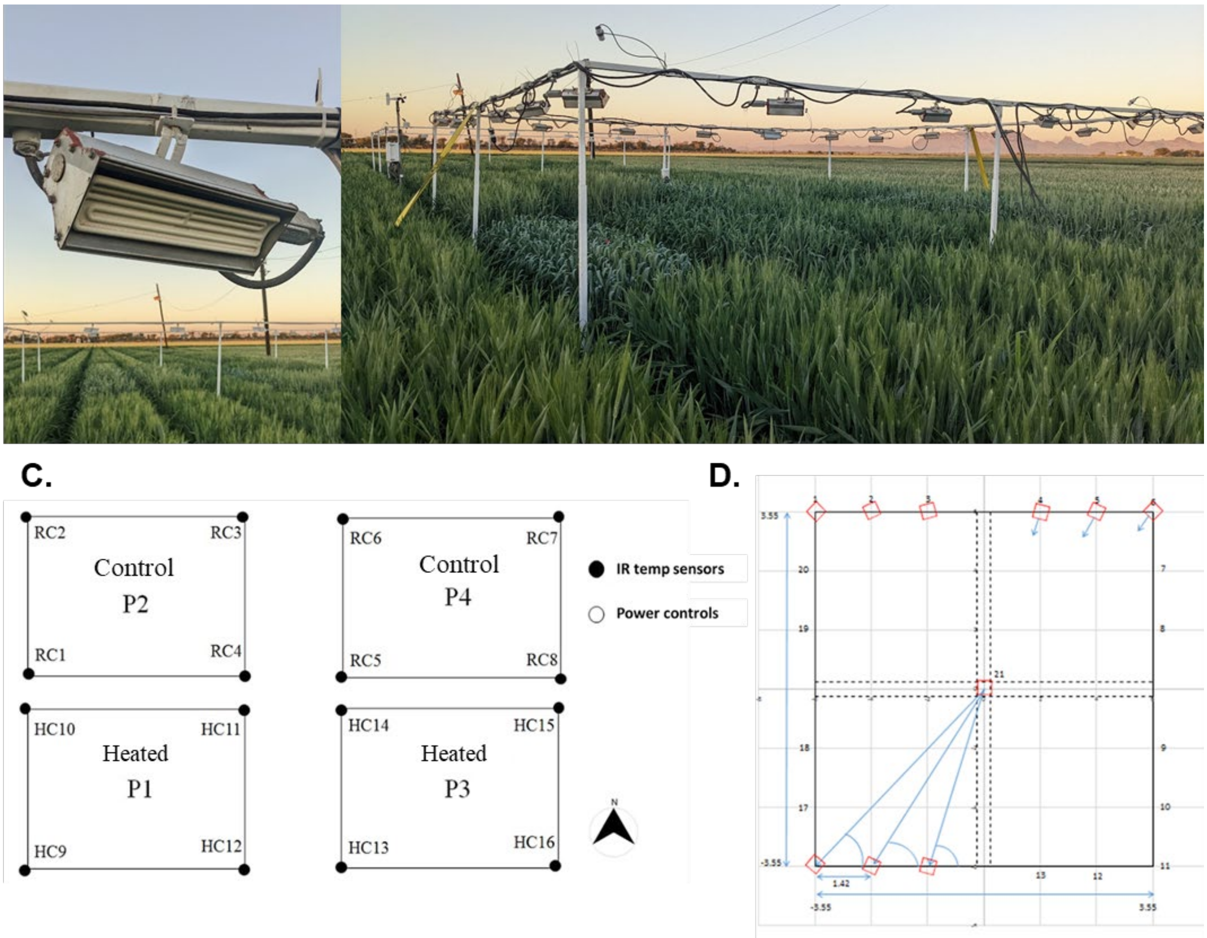

Fig. S1. A close-up of the infra-red heaters installed in the field (a) to artificially elevate air temperature around the treatment plots by 2 °C at night. Placement of heaters in the field experiment (b); a total of 20 heaters were placed along each side and corners of the block of plants under the nocturnal heat treatment plus one additional heater that was hung through the centre of the block, to ensure and maintain an elevated temperature of 2 °C at night. Field experimental design with two blocks of heated and two blocks of control plots (c - P1,P2,P3 and P4). In the corners of each block RC and HC show the location of the IR temperature sensors. Spatial distribution of the 21 heaters placed on each of the four blocks represented with red squares (d).

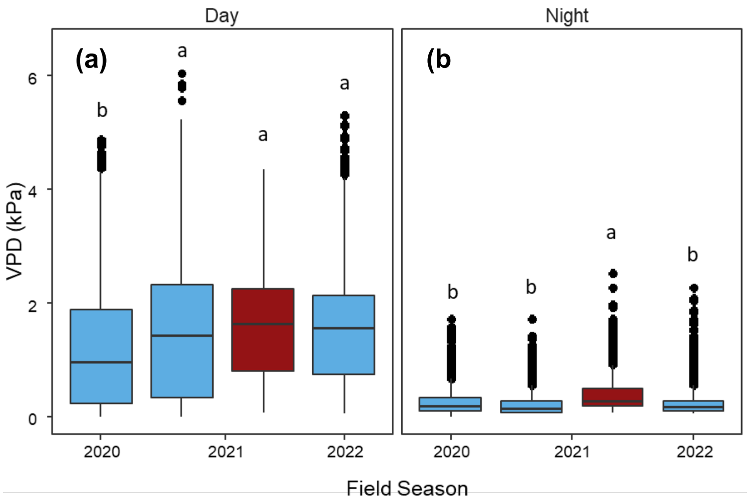

Fig. S2 Vapour pressure differential (VPD) across all three field seasons (2020, 2021, and 2022) as measured during the (a) day and (b) night. All regular sowing experiments (blue) were sown on December 14 or 13 in 2019, 2020, and 2021. The late sowing experiment (red) during the 2021 field season was sown on January 26, 2021. Statistical differences are shown between boxplots as letters where the threshold for a significant difference is $P< 0.05$. The lower and upper borders of the boxplots correspond to the first and third quartiles of the data, the black lines within the boxes indicate the median. Outliers which fall outside the whiskers are shown as black dots (•).

Table S2. Percentage of nights and recorded canopy temperature differences between the heated and control plots each of the three years of experiments. Calculations include the differences starting from the date when the heaters were turned on to the average physiological maturity each season. T_{diff}: nocturnal average canopy temperature of the heated plots minus the average canopy temperature of the control plots.

	% of nights		
**Tdiff (°C)**	2020	2021	2022
≥ 1.8	82	86	94
< 1.8 & ≥ 1.5	8	6	2
< 1.5 & ≥ 1.0	4	4	0
< 1	6	4	4

Table S3. Means and standard deviations for the canopy temperature (CT, °C), relative humidity (RH, %), vapour pressure deficit (VDP, kPa) and the difference between heat and control CT (Tdiff, °C) recorded in the three years of experiments for the whole crop season. Data represents the averages by stage calculated as follows: booting, from start-up of the heating system to the average booting date of each year; heading, from the day after average booting to the average heading date of each year; and grain filling, from the day after average heading to the average physiological maturity of each year. Data was averaged across the 12 genotypes by Time × Year × Treatment.

Time	Year	Booting<br>Control			Heat			
	2020	CT	RH	VPD	CT	RH	VPD	Tdiff
Day		19.8 ± 3.9	83.6 ± 1.8	0.46 ± 0.35	19.6 ± 3.8	85.5 ± 10.6	0.41 ± 0.32	-0.20
Night		14.3 ±2.1	90.7 ± 5.4	0.19 ± 0.13	15.2 ± 3.0	85.7 ± 2.6	0.30 ± 0.07	0.92
	2021							
Day		18.4 ± 6.1	68.5 ± 22.3	1.07 ± 1.0	18.8 ± 6.0	64.6 ± 21.1	1.21 ± 0.95	0.38
Night		9.2 ± 3.4	91.5 ± 8.0	0.13 ± 0.19	11.6 ± 3.3	85.8 ± 6.3	0.23 ± 0.14	2.34
	2022							
Day		16.5 ± 7.5	63.4 ± 18.6	0.98 ± 0.66	16.8 ± 7.3	60.1 ± 18.5	1.10 ± 0.67	0.27
Night		6.7 ± 3.5	89.3 ± 7.2	0.14 ± 0.15	8.4 ± 3.8	87.2 ± 7.4	0.18 ± 0.15	1.67
		Heading<br>Control			Heat			
	2020	CT	RH	VPD	CT	RH	VPD	
Day		20.5 ± 5.5	68.3 ± 22.5	0.97 ± 0.80	20.6 ± 5.4	68.0 ± 20.6	0.97 ± 0.79	0.12
Night		12.6 ±3.4	84.1 ± 10.1	0.29 ± 0.20	14.5 ± 3.9	90.4 ± 8.1	0.17 ± 0.17	1.93
	2021							
Day		18.2 ± 6.24	67.8 ± 20.1	0.99 ± 0.85	18.6 ± 6.2	62.5 ± 19.2	1.19 ± 0.77	0.43
Night		9.4 ± 3.5	89.6 ± 10.2	0.15 ± 0.19	11.6 + 3.5	84.2 ± 9.0	0.25 ± 0.15	2.12
	2022							
Day		16.6 ± 6.3	64.7 ± 15.2	1.01 ± 0.59	16.9 ± 6.1	64.8 ± 18.0	0.98 ± 0.66	0.31
Night		8.1 ± 3.1	90.0 ± 6.0	0.14 ± 0.14	10.3 ± 3.0	86.1 ± 6.5	0.20 ± 0.13	2.20
		Grainfilling<br>Control			Heat			
	2020	CT	RH	VPD	CT	RH	VPD	
Day		21.6 ± 6.5	67.7 ± 18.2	1.20 ± 0.63	22.1 ± 6.5	65.4 ± 17.0	1.27 ± 0.63	0.42
Night		12.7 ± 4.1	90.9 ± 5.6	0.17 ± 0.12	14.5 ± 3.2	83.8 ± 4.9	0.31 ± 0.12	1.86
	2021							
Day		22.7 ± 7.6	61.1 ± 22.8	1.59 ± 1.3	22.9 ± 7.3	55.5 ± 23.3	1.82 ± 1.28	0.15
Night		10.4 ± 4.5	89.9 ± 9.2	0.17 ± 0.26	12.6 ± 4.3	84.3 ± 8.0	0.282 ± 0.21	2.10
	2022							
Day		21.5 ± 7.3	54.0 ± 17.5	1.62 ± 0.75	22.0. ± 7.3	53.2 ± 19.7	1.66 ± 0.78	0.48
Night		10.0 ± 4.7	88.1 ± 8.0	0.20 ± 0.19	12.0 ± 4.4	83.6 ± 9.0	0.28 ± 0.18	2.02

Table S4. Average days to initiation of booting (DTB), days to heading (DTH), days to anthesis (DTA), days to maturity (DTM), and days to 75% grain filling (A+75%GF) across years and treatments.

Year	Treatment	DTB	DTH	DTA	DTM	A+75%GF
2020	Control	59	69	74	112	103
	Nocturnal Heat	59	68	73	111	101
2021	Control	65	77	82	116	108
	Nocturnal Heat	64	76	81	115	106
	Late Sowing	59	61	NA	92	NA
2022	Control	60	72	77	118	108
	Nocturnal Heat	60	72	77	116	107

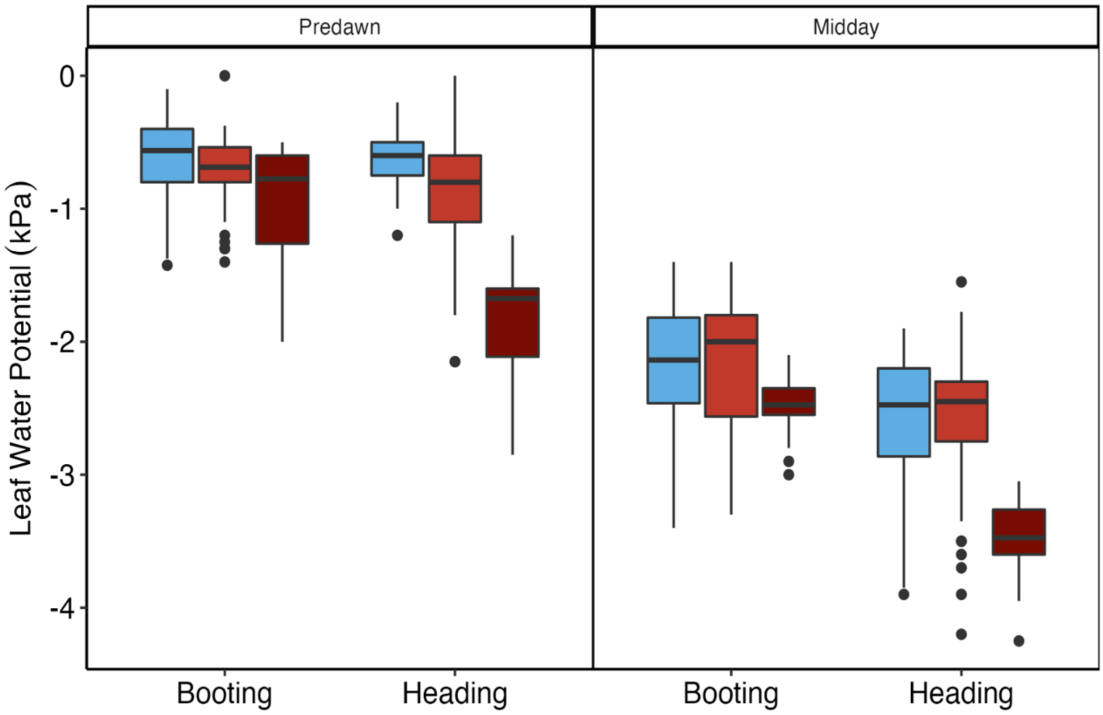

Fig. S3 Water potential measured predawn and at midday for leaves in booting or heading plants. Regular sown plants were subject to control (blue) or nocturnal heating (light red). Measurements were also made on plants which were late sown (dark red). The data shown are the cumulative responses of 12 genotypes over three years of measurement (2020-2022) with two biological replicates grown in each treatment per year. The lower and upper borders of the boxplots correspond to the first and third quartiles of the data, the black lines within the boxes indicate the median. Outliers which fall outside the whiskers are shown as black dots (•).

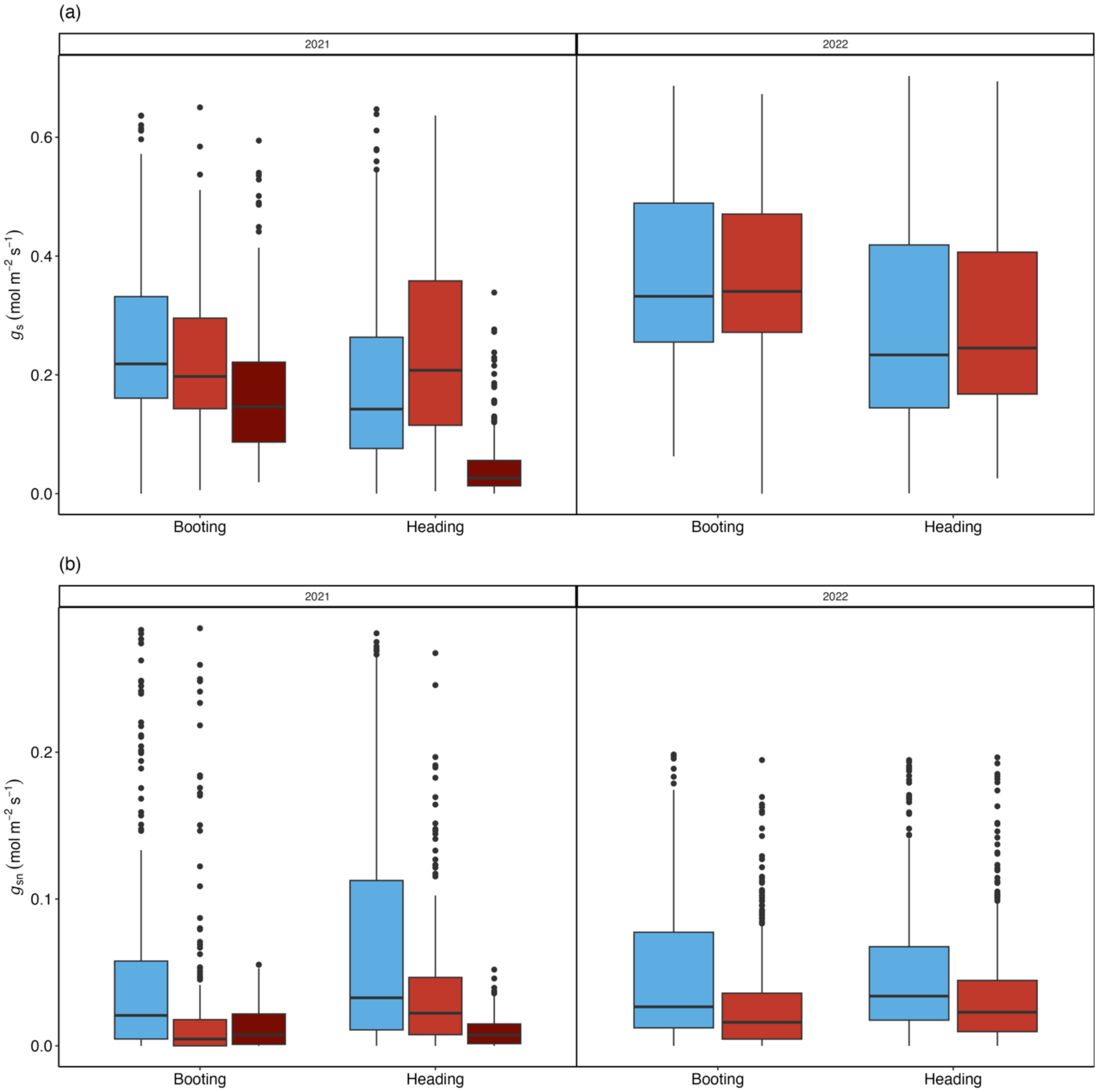

Fig. S4 The response of stomatal conductance, measured during the day (a – g_s) or at night (b – g_{sn}) in booting or heading plants in 2021 and 2022. Regular sown plants were subject to control (blue) or nocturnal heating (light red). Measurements were also made on plants which were late sown (dark red) in 2021. The data shown are the cumulative responses of twelve genotypes with two biological replicates grown in each treatment per year. The lower and upper borders of the boxplots correspond to the first and third quartiles of the data, the black lines within the boxes indicate the median. Outliers which fall outside the whiskers are shown as black dots (•).

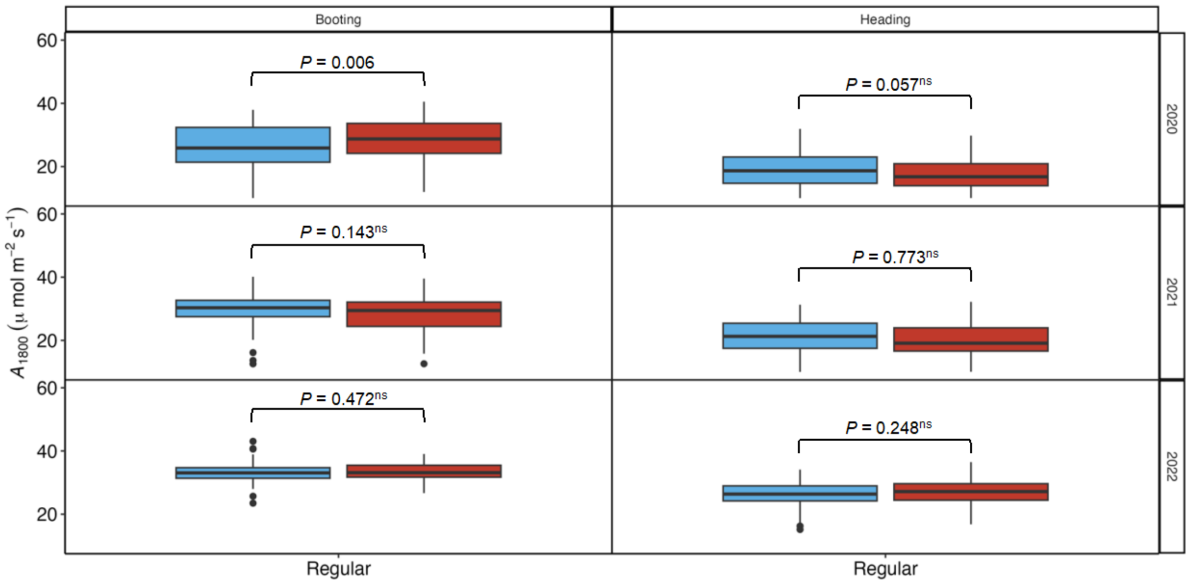

Fig. S5 The response of CO_2 assimilation (*A*) under 1800 µmol m^{-2} s^{-1} photosynthetic photon flux density (PPFD - A_{1800}) for 12 genotypes under control (blue) or nocturnally heated (red) plots over three years (2020-2022). The data is collected from the regular sowing only. Measurements are individual data points (n = 2 biological replicates per year per treatment). The lower and upper borders of the boxplots correspond to the first and third quartiles of the data, the black lines within the boxes indicate the median. Outliers which fall outside the whiskers are shown as black dots (•).

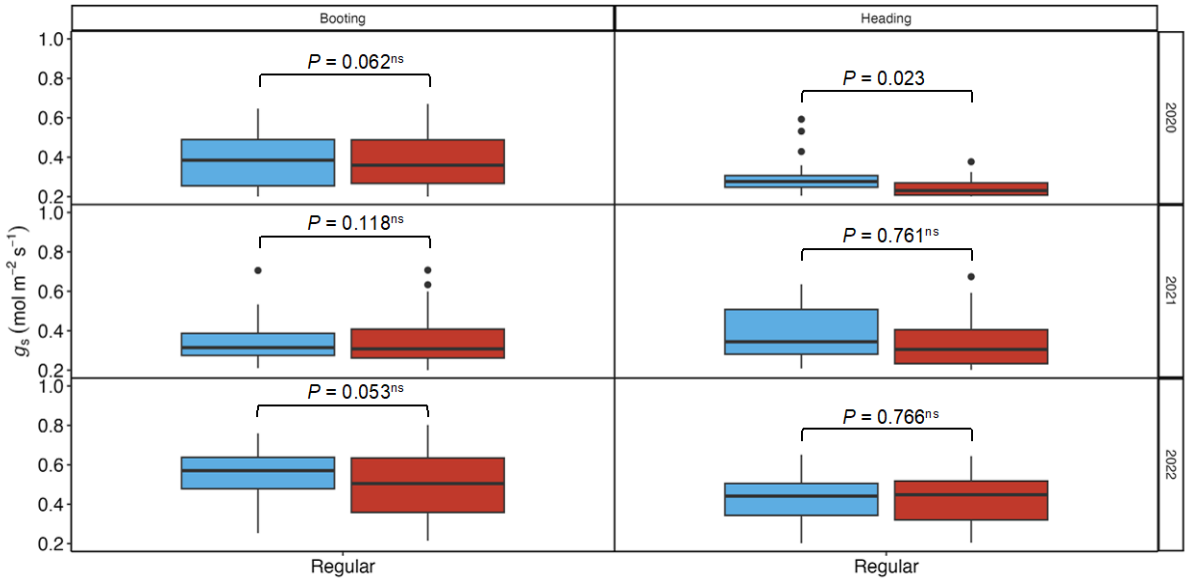

Fig. S6 The response of daytime stomatal conductance (g_s) at 1800 µmol m^{-2} s^{-1} photosynthetic photon flux density (PPFD) for 12 genotypes under control (blue) or nocturnally heated (red) plots over three years (2020-2022). The data is collected from the regular sowing only. Measurements are individual data points (n = 2 biological replicates per year per treatment). The lower and upper borders of the boxplots correspond to the first and third quartiles of the data, the black lines within the boxes indicate the median. Outliers which fall outside the whiskers are shown as black dots (•).

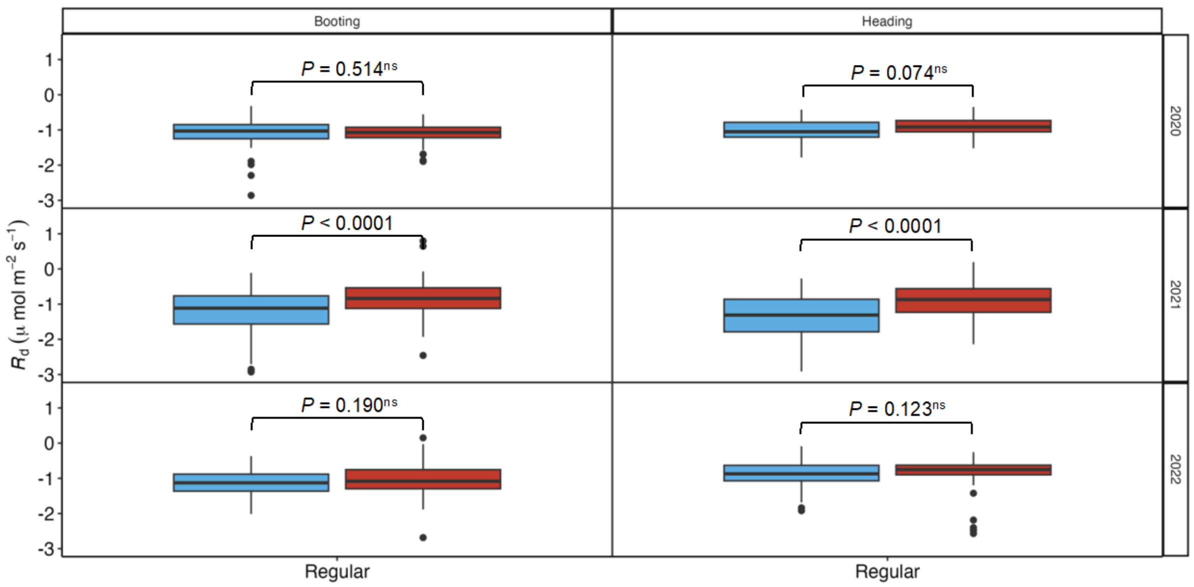

Fig. S7 The response of nocturnal respiration (R_d) for 12 genotypes under control (blue) or nocturnally heated (red) plots over three years (2020-2022). The data is collected from the regular sowing only. Measurements are individual data points (n = 2 biological replicates per year per treatment). The lower and upper borders of the boxplots correspond to the first and third quartiles of the data, the black lines within the boxes indicate the median. Outliers which fall outside the whiskers are shown as black dots (•).

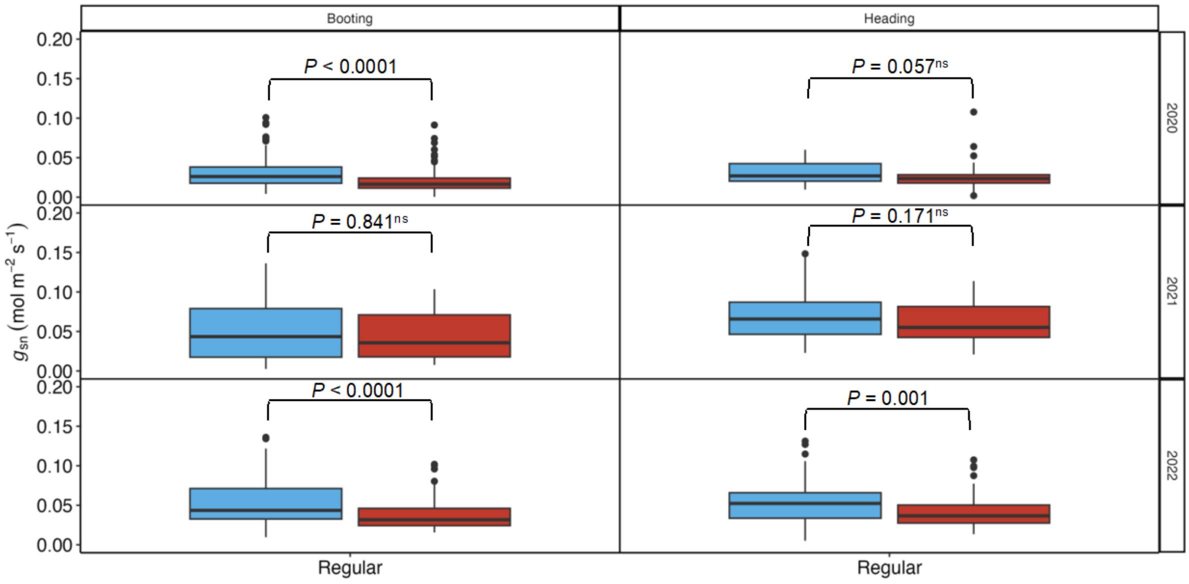

Fig. S8 The response of nocturnal stomatal conductance (g_{sn}) for 12 genotypes under control (blue) or nocturnally heated (red) plots over three years (2020-2022). The data is collected from the regular sowing only. Measurements are individual data points (n = 2 biological replicates per year per treatment). The lower and upper borders of the boxplots correspond to the first and third quartiles of the data, the black lines within the boxes indicate the median. Outliers which fall outside the whiskers are shown as black dots (•).

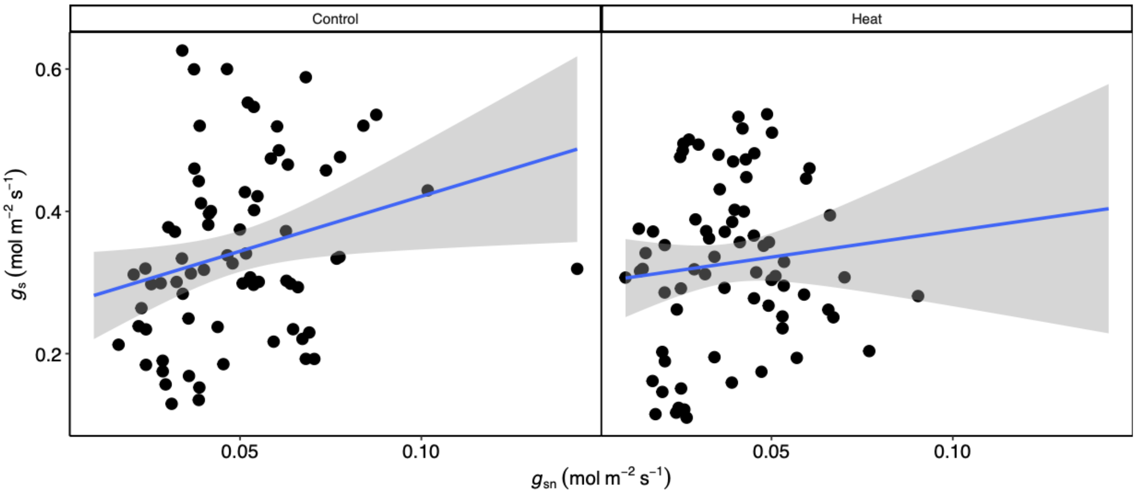

Fig S9 Correlating daytime stomatal conductance (g_s) values with nocturnal conductance values under control or heated nocturnal conditions. Data are the means for each genotype over three years of measurement (n= 3-17). A regression line indicates the strength of correlation, while the shaded grey region indicates 95% confidence interval.

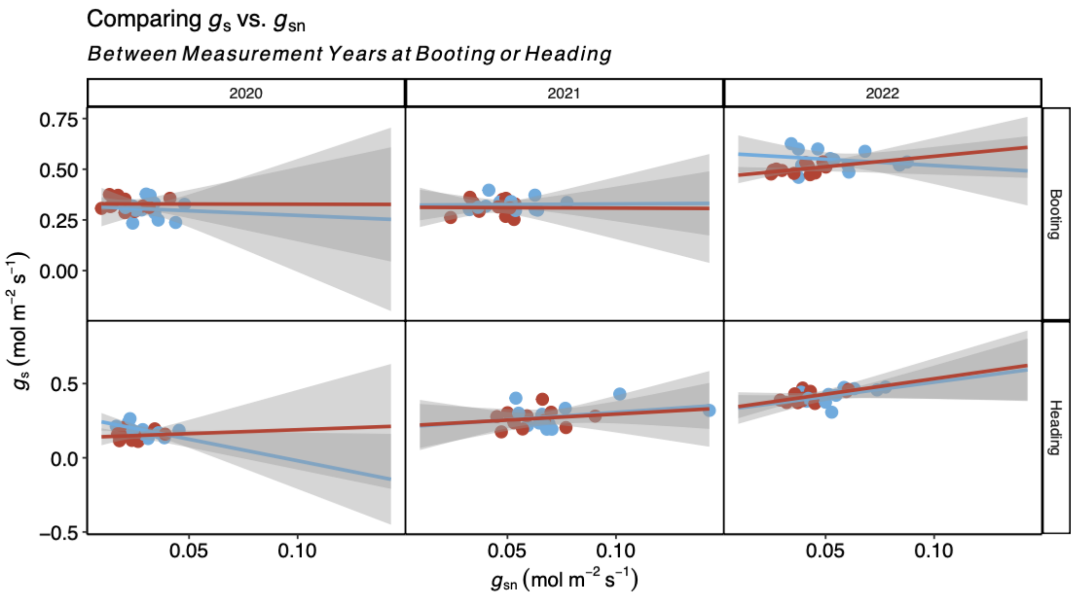

Fig. S10 Comparing the response of daytime stomatal conductance (*g*s) and nocturnal stomatal conductance (*g*sn) between the measurement years (2020-2022) and at two growth stages - booting and heading. Data are the means for each genotype measured (n = 3-17), measured under control conditions (blue) or under nocturnal heat (red). A regression line indicates the strength of correlation, while the shaded grey region indicates 95% confidence interval.

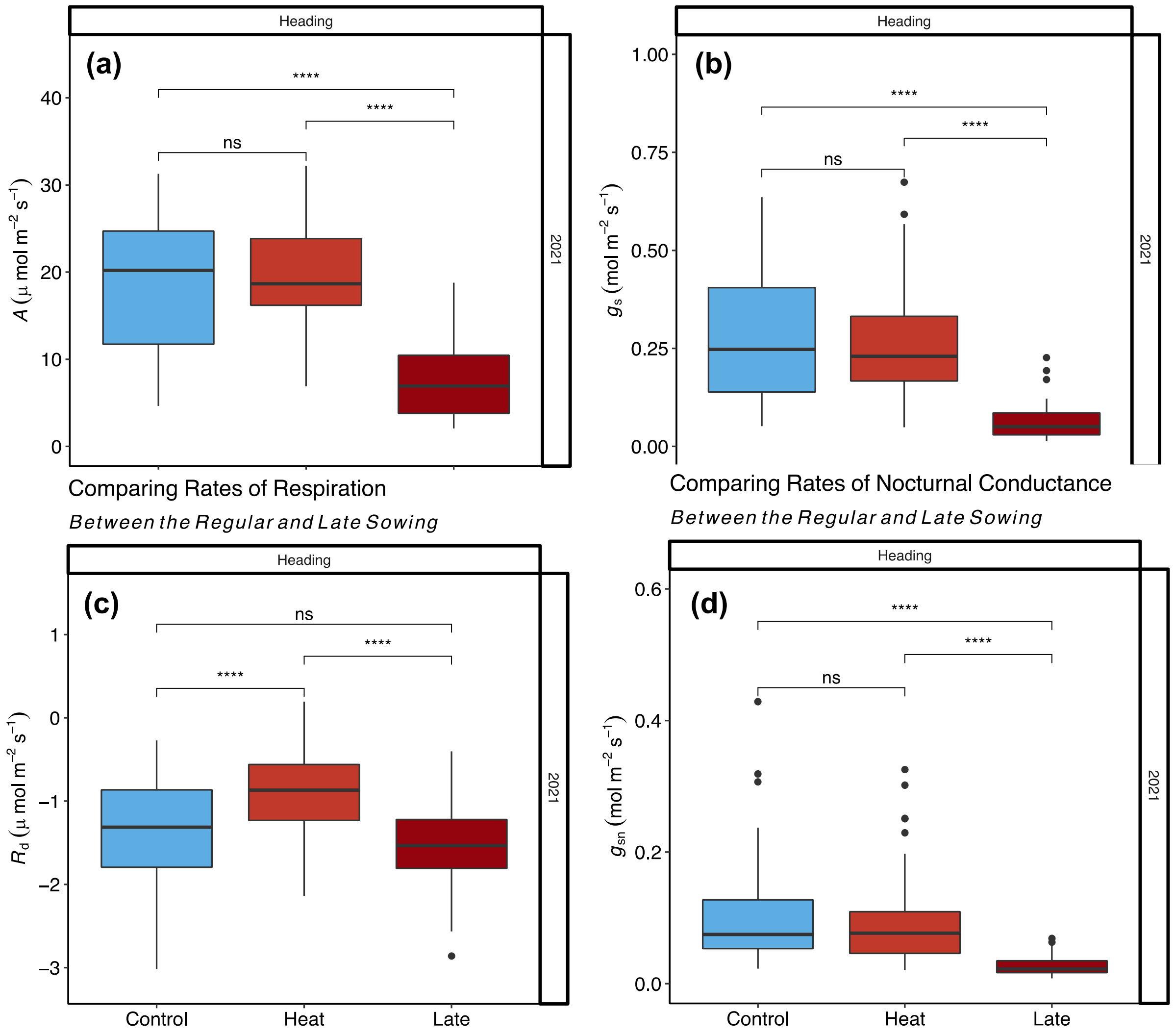

Fig. S11 Comparing the mean response of CO_2 assimilation (a - A), daytime stomatal conductance (b - g_s), nocturnal respiration (c- R_d) and nocturnal stomatal conductance (d - g_{sn}) between the regular sown plants under either control (blue) or nocturnal heating (red), with the response of the late sown plants (dark red).

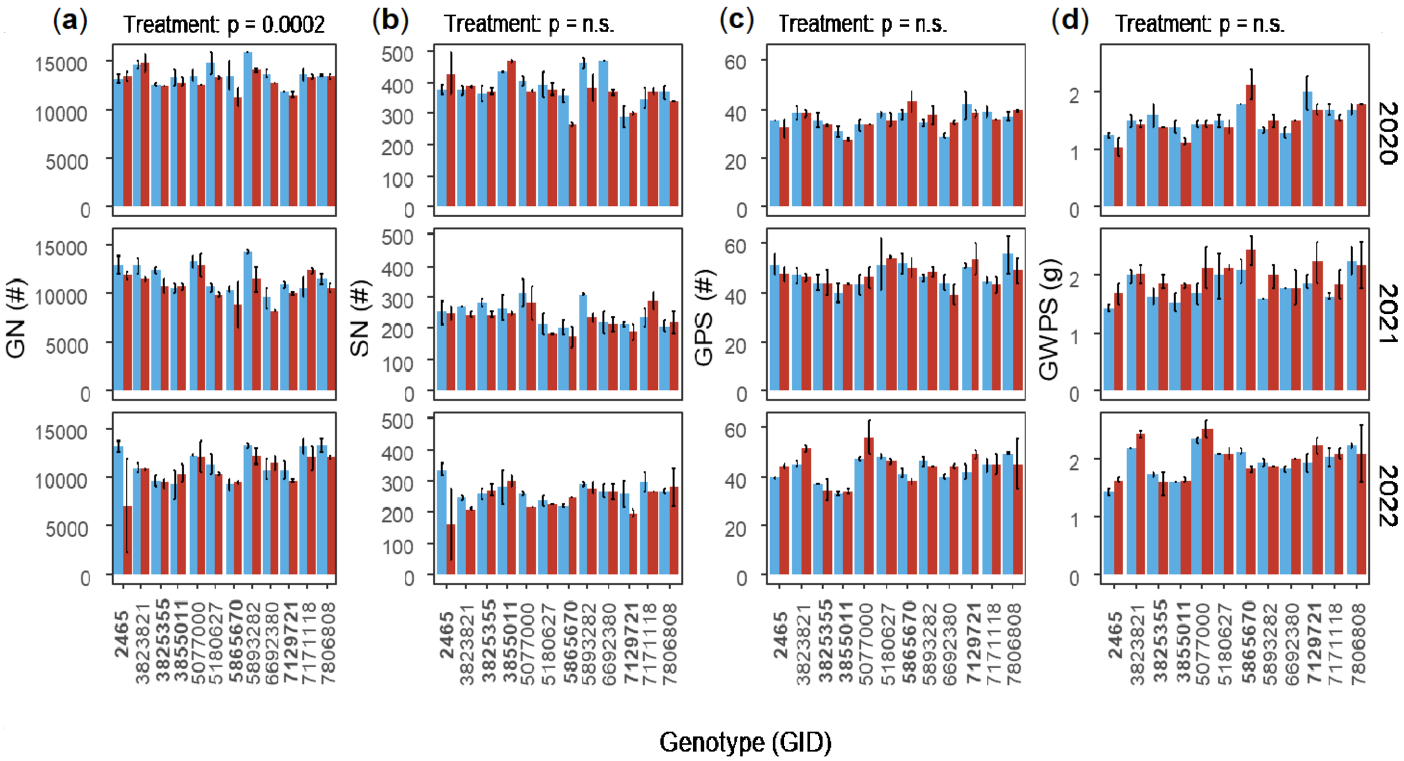

Fig. S12. Distribution of grain number (a - GN), spike number (b - SN), grain per spike (c - GPS), and grain weight per spike (d- GWPS) across the three measured field seasons (2020, 2021, 2022), two treatments (yield potential in blue, heat treatment in red), and in the twelve selected genotypes. Genotypes in bold are those which were predetermined as “heat tolerant”. Error bars represent standard error (SE) for each genotype and treatment.

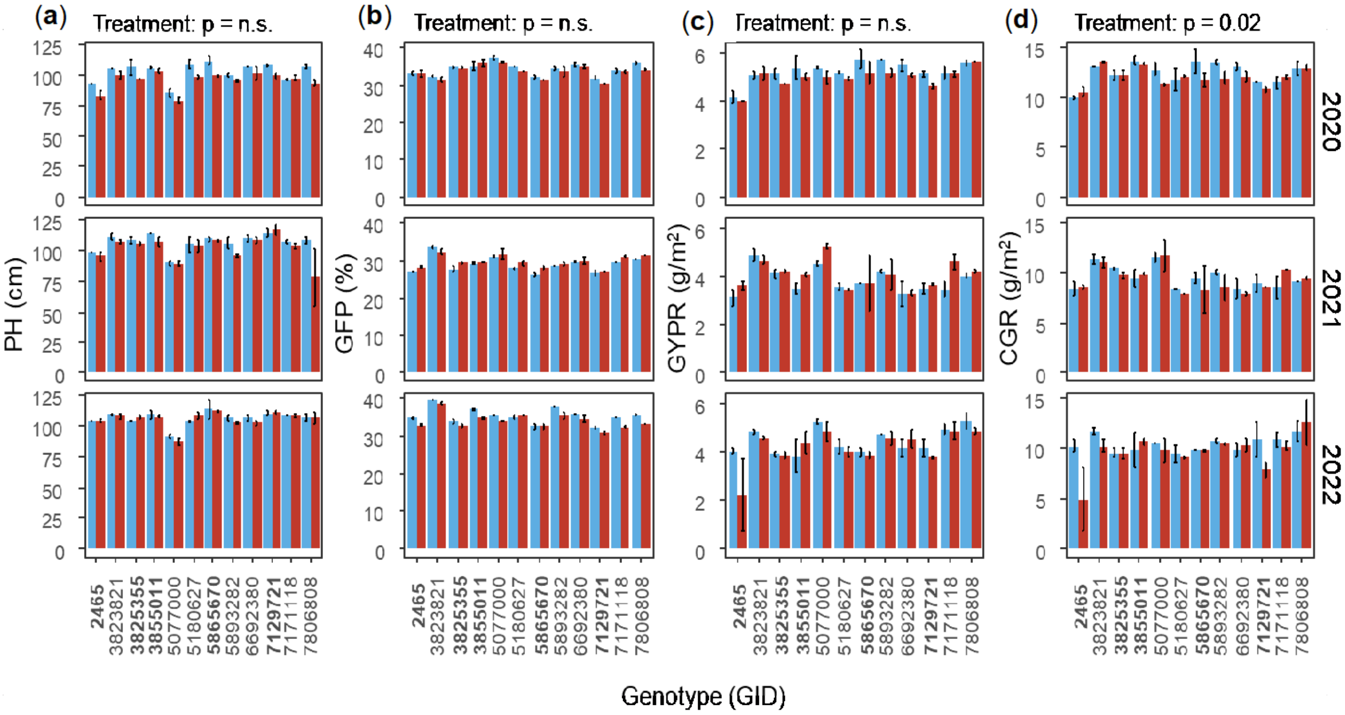

Fig. S13. The distribution of plant height (a - PH), grain filling period (b - GFP), grain yield production rate (c -GYPR), and crop growth rate per day (d - CGR) across the three measured field seasons (2020, 2021, 2022), two treatments (yield potential in blue, heat treatment in red), and in the twelve selected genotypes. Genotypes in bold are those which were predetermined as “heat tolerant”. Error bars represent standard error (SE) for each genotype and treatment. per day
